# Supplementary figures and images for: Poststroke dendritic arbor regrowth requires the actin nucleator Cobl
Source: PLoS Biol. 2021 Dec 13;19(12):e3001399. doi: 10.1371/journal.pbio.3001399 (PMC8699704; doi:10.1371/journal.pbio.3001399)

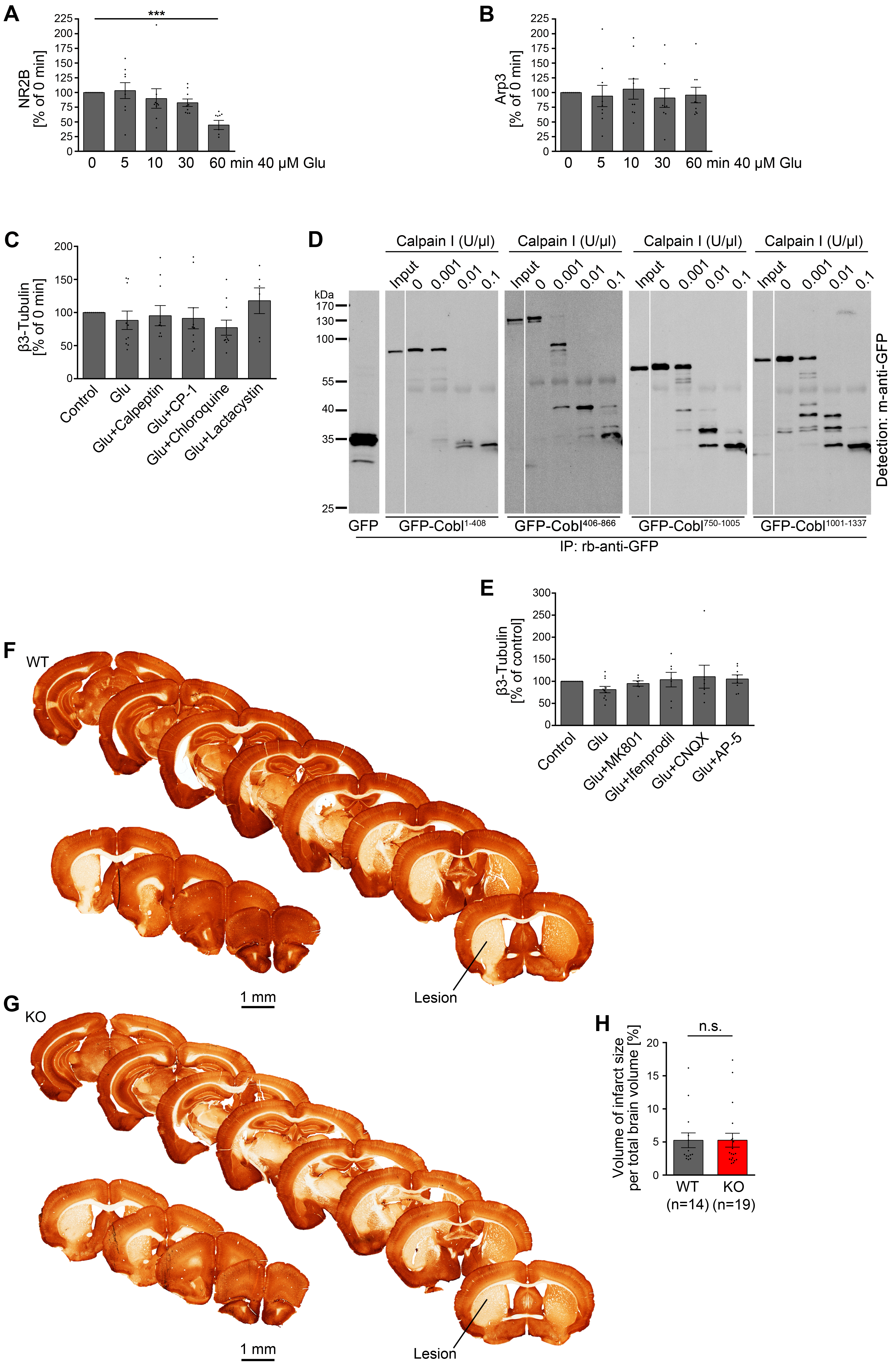

Supplement: S1 Fig — The actin nucleator Cobl is degraded by the Ca2+-controlled protease calpain during NMDAR-mediated excitotoxicity but Cobl KO does not affect the size of the final lesion caused by ischemic stroke induced by MCAO. A, B, Quantitative immunoblotting analyses of NR2B (A) and Arp3 (B) in cortical neuronal cultures subjected to different durations of incubation with 40 μM glutamate (Glu). n = 9 independent assays and biological samples. C, Proteolytic pathways underlying the Cobl decline upon prolonged stimulation with glutamate, as shown by the use of inhibitors against calpain (Calpeptin, CP-1), against lysosomal degradation (Chloroquine) and aginst proteasomal proteolysis (Lactacystin), respectively, in quantitative immunoblotting analyses. nControl = 10, nGlu = 10, nGlu+Calpeptin = 10, nGlu+CP-1 = 10, nGlu+Chloroquine = 10, nGlu+Lactacystin = 6 independent biological samples. D, Anti-GFP immunoblotting analyses of GFP-Cobl1-408, GFP-Cobl406-866, GFP-Cobl750-1005, and GFP-Cobl1001-1337 expressed in HEK293 cells, immunoisolated with anti-GFP antibodies and incubated without and with calpain I (10 minutes, 25°C). Input shows the GFP fusion protein prior to the incubation with calpain concentrations ranging from 0 to 0.1 U/μl. GFP is shown for size comparison. White lines indicate lanes omitted from the blots. Size standards apply to all blots shown. E, Lack of effects of inhibitors against open NMDARs (Glu+MK801), against the NR2B subunits of NMDA receptors (Glu+Ifenprodil), against AMPA and kainate receptors (Glu+CNQX), and against NMDARs (Glu+AP-5), respectively, when compared to control and glutamate-induced (30 minutes, 40 μM) excitotoxicity in quantitative anti-β3-tubulin immunoblotting analyses of lysates of neuronal cultures. ncontrol = 11, nGlu = 11, nGlu+MK-801 = 7, nGlu+Ifenprodil = 7, nGlu+CNQX = 7, nGlu+AP-5 = 8 independent biological samples. Statistical significance calculations, 1-way ANOVA with Dunn posttest (A–C, E). ***P < 0.001. F,G, Representative ex [file pbio.3001399.s001.tif]

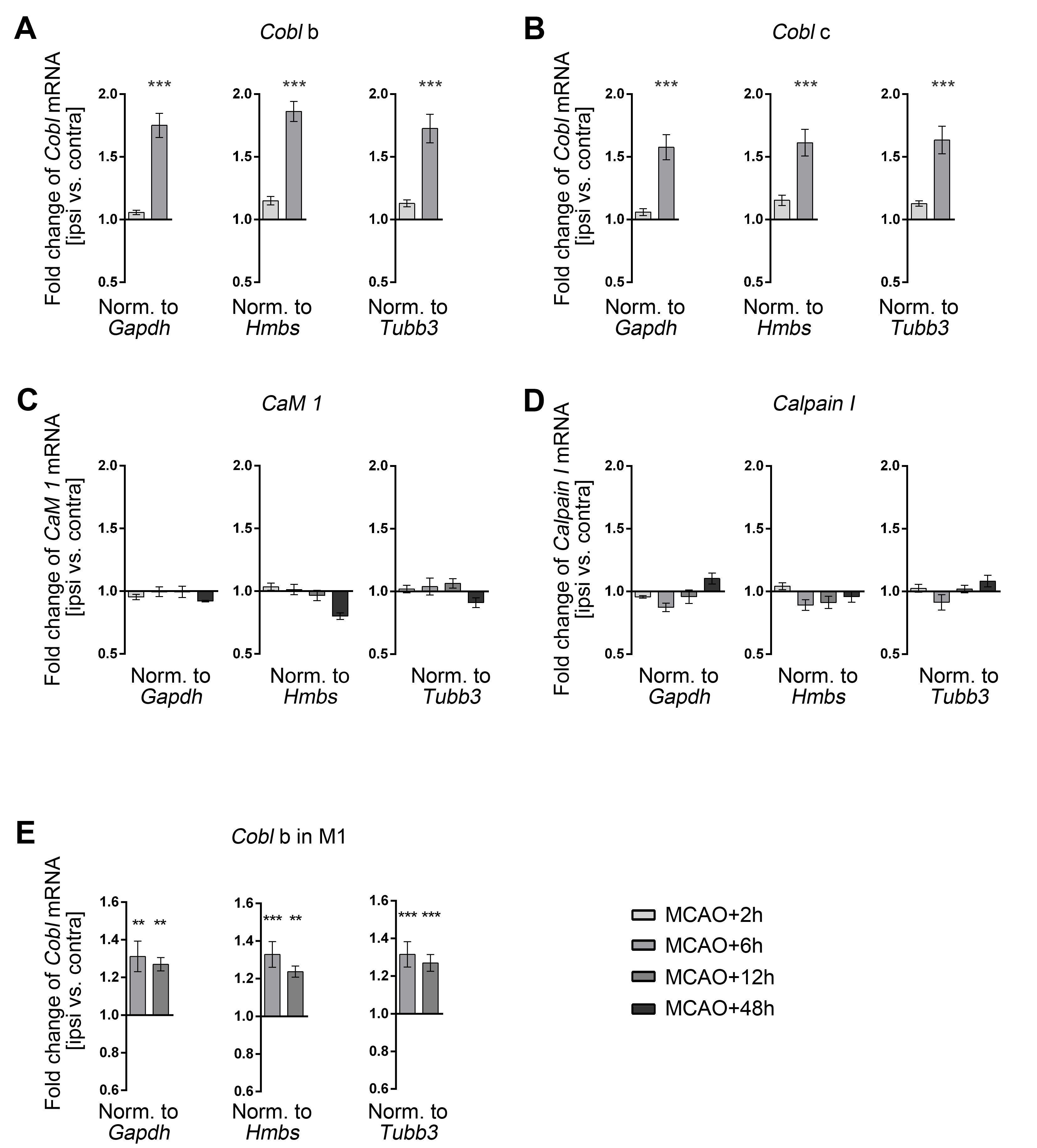

Supplement: S2 Fig — qPCR analyses demonstrate a transient excess of ipsilateral Cobl mRNA expression 6 hours after MCAO. A,B, Fold change of Cobl mRNA at 2 hours and 6 hours reperfusion after MCAO, as determined by qPCR with Cobl b (A) and Cobl c (B) primers, respectively. qPCR data represent the differences between ipsi and contra of Cobl (primer sets b and c, respectively) normalized to Gapdh (left panel), Hmbs (middle panel), and Tubb3 (right panel), respectively. n MCAO+2h = 8; n MCAO+6h = 8 brain samples and mice. C,D, Fold change of CaM 1 (C) and Calpain I (D) mRNA levels at 2 hours, 6 hours, 12 hours, and 48 hours reperfusion time after 30-minute MCAO. Data represent ratios of the differences between ipsi and contra of CaM 1 and Calpain I levels, respectively, normalized to Gapdh, Hmbs, and Tubb3, respectively. E, Fold changes of Cobl mRNA levels in M1 tissue samples (ipsi versus contra) 6 hours and 12 hours after MCAO. The data are again normalized against 3 different genes (Gapdh, Hmbs, and Tubb3). A–D, n MCAO+2h = 8; n MCAO+6h = 8; n MCAO+12h = 9; n MCAO+48h = 6 brain samples and mice. E, nMCAO+6h = 7; nMCAO+12h = 8 M1 samples and mice. Data, mean ± SEM. Statistical significances (ipsi versus contra) were calculated using 1-way ANOVA with Sidak posttest (A–E), respectively. **P < 0.01; ***P < 0.001. The numerical data underlying this figure can be found in S9 Data. MCAO, middle cerebral artery occlusion; qPCR, quantitative PCR. (TIF) [file pbio.3001399.s002.tif]

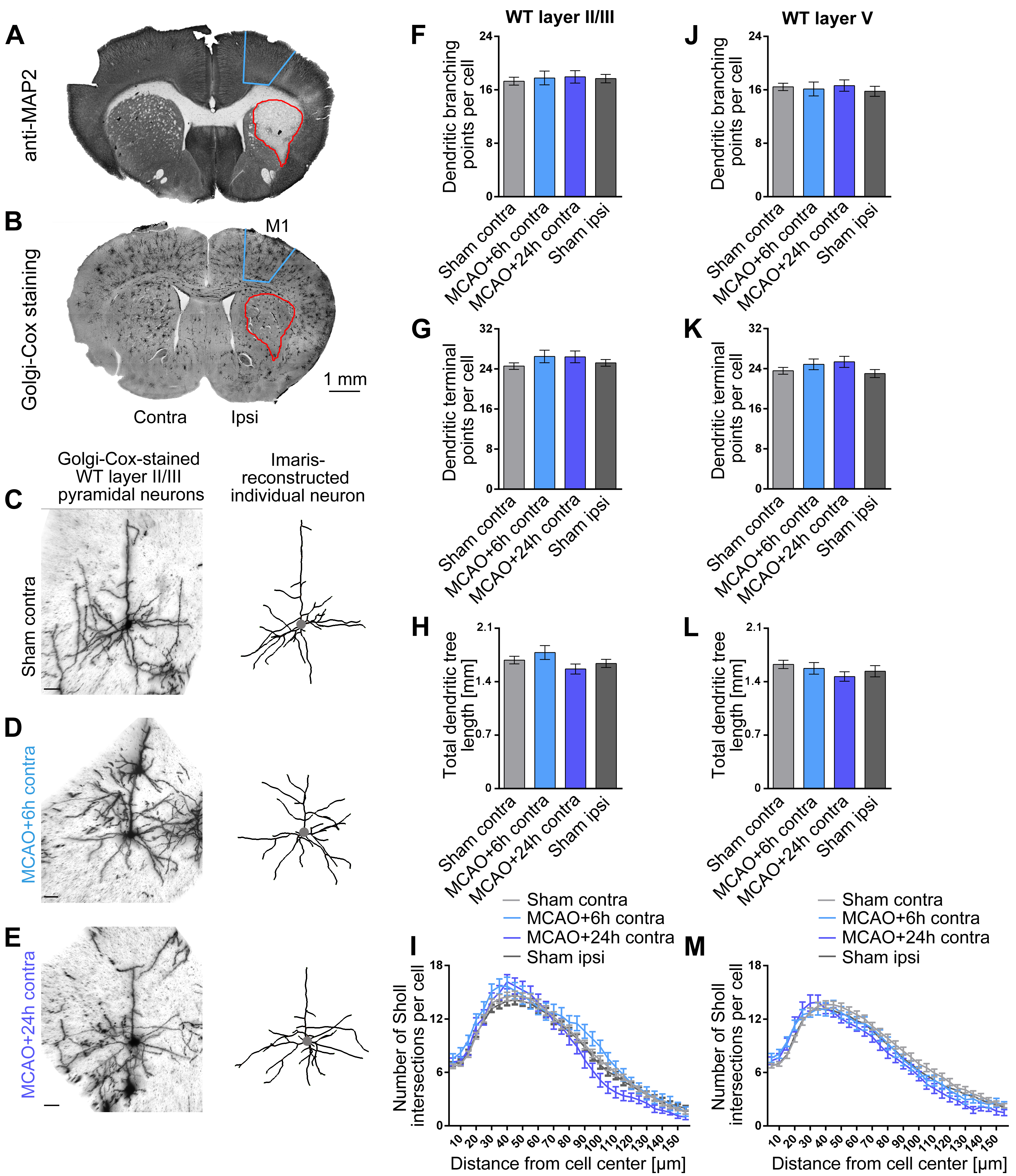

Supplement: S3 Fig — No change in dendritic arbor complexity subsequent to ischemic stroke in layer II/III and layer V neurons in the contralateral motor cortex. A,B, Representative micrographs of adjacent serial coronal sections of brains at ~bregma +0.8 mm from WT mice (age, 3 to 4 months) that were subjected to 30-minute MCAO. A, Anti-MAP2 immunostaining. B, Golgi–Cox staining. The lesion caused by the infarct can be determined based on the lack of anti-MAP2 detection (outlined in red). Blue framing indicates the adjacent region M1 used for morphological analysis in corresponding Golgi–Cox-stained sections. C-E, Representative images of Golgi–Cox-stained (left panels) and Imaris-reconstructed (right panels) layer II/III pyramidal neurons of M1 from the contralateral side of sham-treated mice (C) and of mice subjected to 30-minute MCAO analyzed after 6 hours (D) and 24 hours (E) reperfusion times. The position of the cell bodies are marked by a gray dot. Scale bars, 30 μm. F–M, Quantitative determinations of dendritic branching points (F, J), dendritic terminal points (G, K), total dendritic tree length (H, L), and Sholl intersections (I, M) of dendritic trees in layer II/III (F–I) and layer V of M1 (J–M). Note that all parameters of dendritic complexity at the contralateral side remained unchanged in comparison to sham-treated mice at both 6 hours and 24 hours after MCAO. Layer II/III: nSham contra = 57; nMCAO+6h contra = 18; nMCAO+24h contra = 17; nSham ipsi = 60 neurons. Layer V: nSham contra = 60; nMCAO+6h contra = 22; nMCAO+24h contra = 25; nSham ipsi = 47 neurons from 3 mice each for the 2 MCAO conditions (for ipsi data from the same mice, see Fig 4) and from 6 mice for sham controls (contralateral and ipsilateral; sham ipsi data as in Fig 4 for comparison). Quantitative data represent mean ± SEM. Statistical significance calculations, 1-way ANOVA with Tukey posttest (F–H, J–L) and 2-way ANOVA with Sidak posttest for Sholl analysis (I, M), respectively (all n.s.). The numerical [file pbio.3001399.s003.tif]

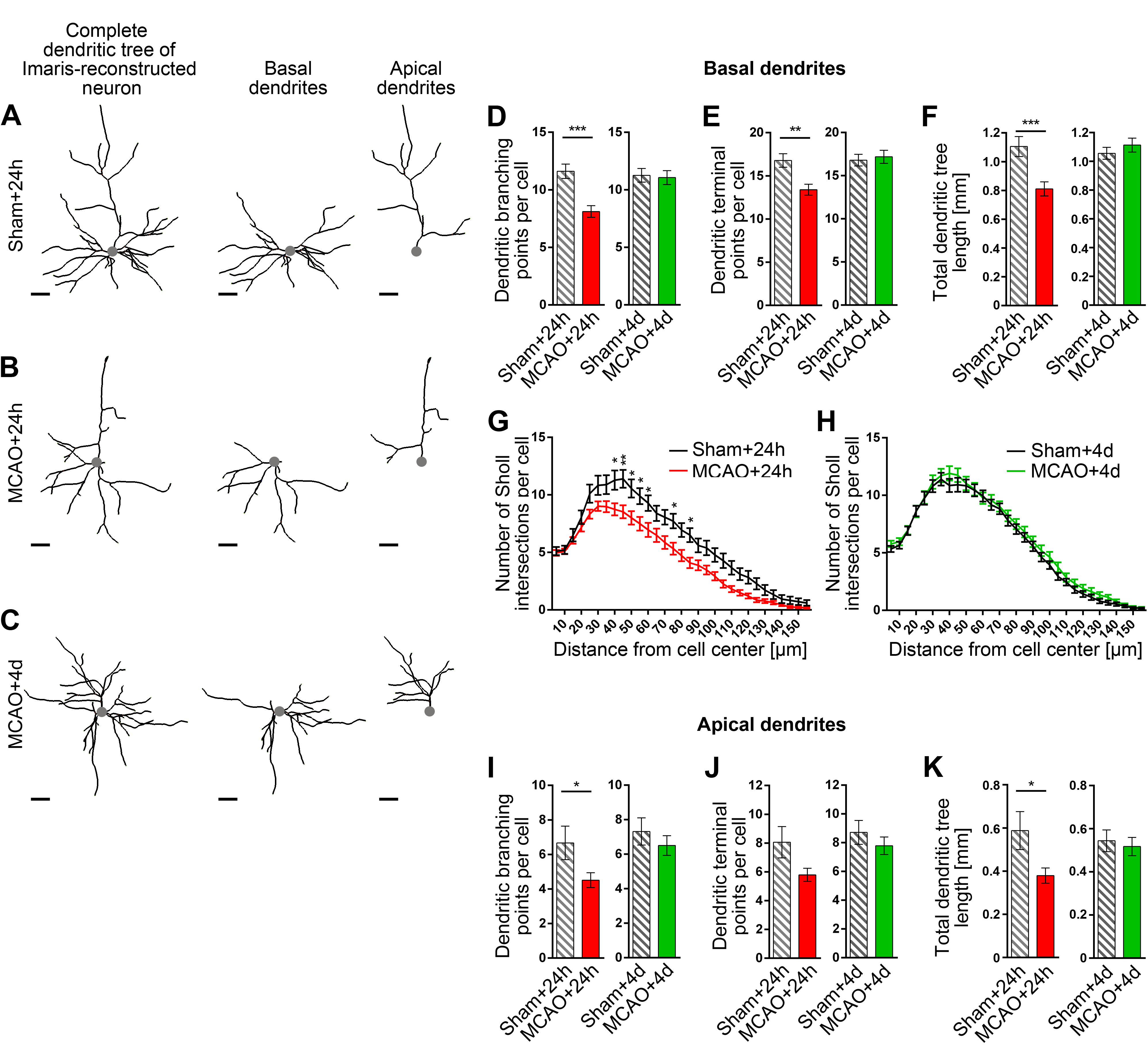

Supplement: S4 Fig — MCAO-induced defects in dendritic arborization manifest in both apical and basal dendrites of layer II/III pyramidal neurons of M1. A–C, Individual Imaris-reconstructed cells from Fig 5A–5C (right panels) showing ipsilateral layer II/III neurons from M1 of WT mice subjected to sham treatment (A) and 30-minute MCAO with 24 hours and 4 days reperfusion time (B, C), respectively, for differential analyses of basal (D–H) and apical dendritic parameters (I–K). The apical and basal dendritic parts are depicted in A–C. The positions of the cell bodies are marked by a gray dot. Scale bars, 30 μm. D–K, Quantitative determinations of dendritic branching points, terminal points, total dendritic tree length, and Sholl intersections of basal dendrites (D–H) and related analyses for apical dendrites (I–K). Note that MCAO-induced defects occur in both apical and basal dendrites 24 after MCAO and also show full recovery in both apical and basal dendritic arbors after 4 days reperfusion. nSham+24h = 18; nMCAO+24h = 43; nSham+4d = 35; nMCAO+4d = 32 neurons from 6 mice for the MCAO+24h group and 3 mice for the MCAO+4d group and from 3 mice for each sham control group (ipsi). Quantitative data represent mean ± SEM. Statistical significance calculations, Mann–Whitney (D–F, I–K) and 2-way ANOVA with Sidak posttest for Sholl analysis (G, H), respectively. *P < 0.05; **P < 0.01; ***P < 0.001. The numerical data underlying this figure can be found in S11 Data. MCAO, middle cerebral artery occlusion; WT, wild-type. (TIF) [file pbio.3001399.s004.tif]

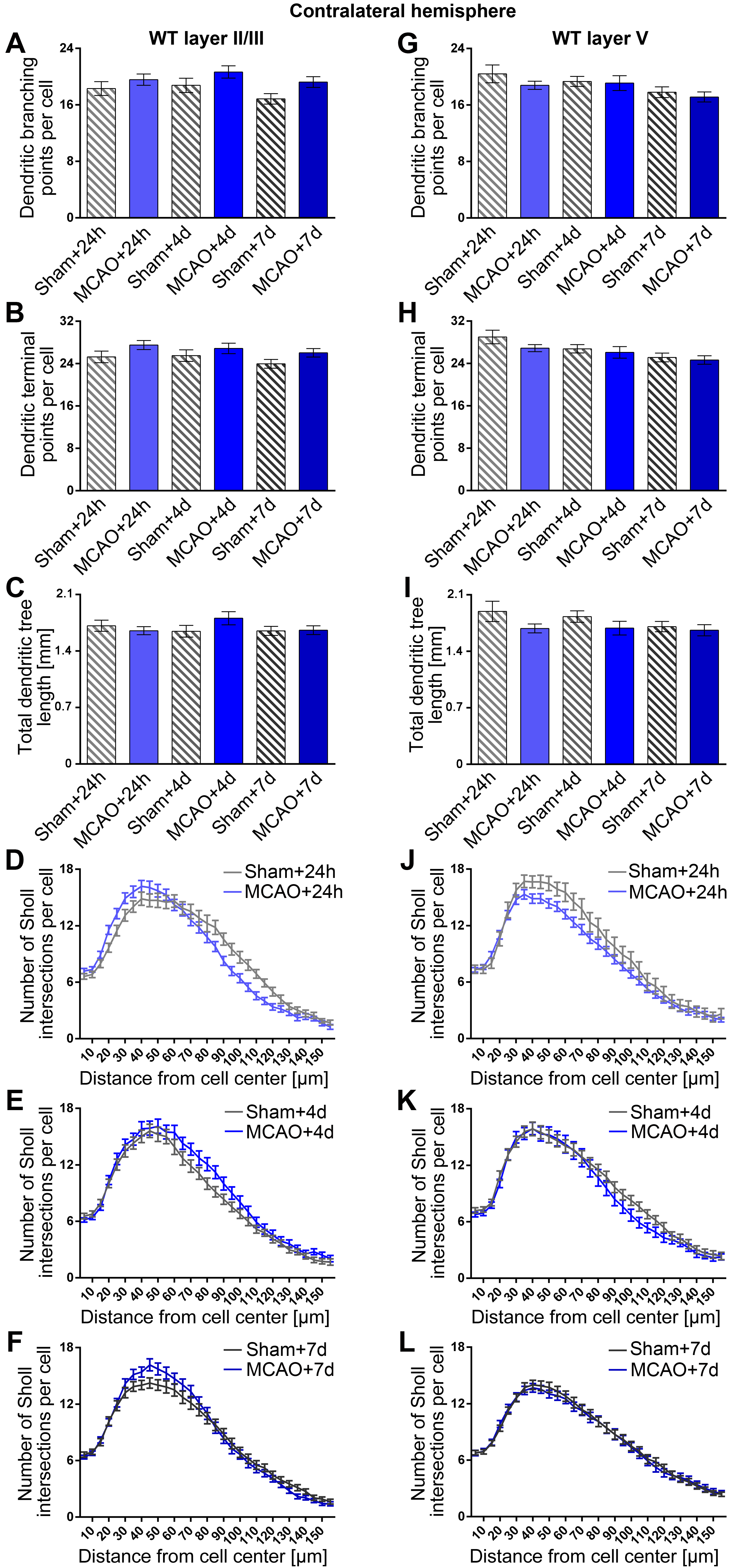

Supplement: S5 Fig — The dendritic arborization of neurons at the contralateral side is not affected by the MCAO-induced dendritic regrowth processes occurring simultaneously at the ipsilateral side. A–L, Quantitative determinations of dendritic arborization parameters of layer II/III neurons (A–F) and layer V neurons (G–L) in the contralateral M1. Note that, at the contralateral side, dendritic branching points (A, G), dendritic terminal points (B, H), total dendritic tree length (C, I) and Sholl intersections (D–F, J–L) at different reperfusion times all remained similar to their respective sham controls and at the same level for all 3 time points (24 hours, 4 days, and 7 days). Layer II/III: nSham+24h = 29; nMCAO+24h = 53; nSham+4d = 38; nMCAO+4d = 29; nSham+7d = 46; nMCAO+7d = 49 neurons from 3 mice for the sham+24h, sham+4d and MCAO+4d groups, and from 6 mice for the sham+7d, MCAO+24h, and MCAO+7d groups (3 to 4 months of age). Layer V: nSham+24h = 17; nMCAO+24h = 57; nSham+4d = 30; nMCAO+4d = 30; nSham+7d = 57; nMCAO+7d = 50 neurons from 3 mice of each group. For corresponding ipsilateral data, see Fig 5. Data, mean ± SEM. Statistical significances were calculated using 2-way ANOVA with Sidak posttest (all n.s.). The numerical data underlying this figure can be found in S12 Data. MCAO, middle cerebral artery occlusion. (TIF) [file pbio.3001399.s005.tif]

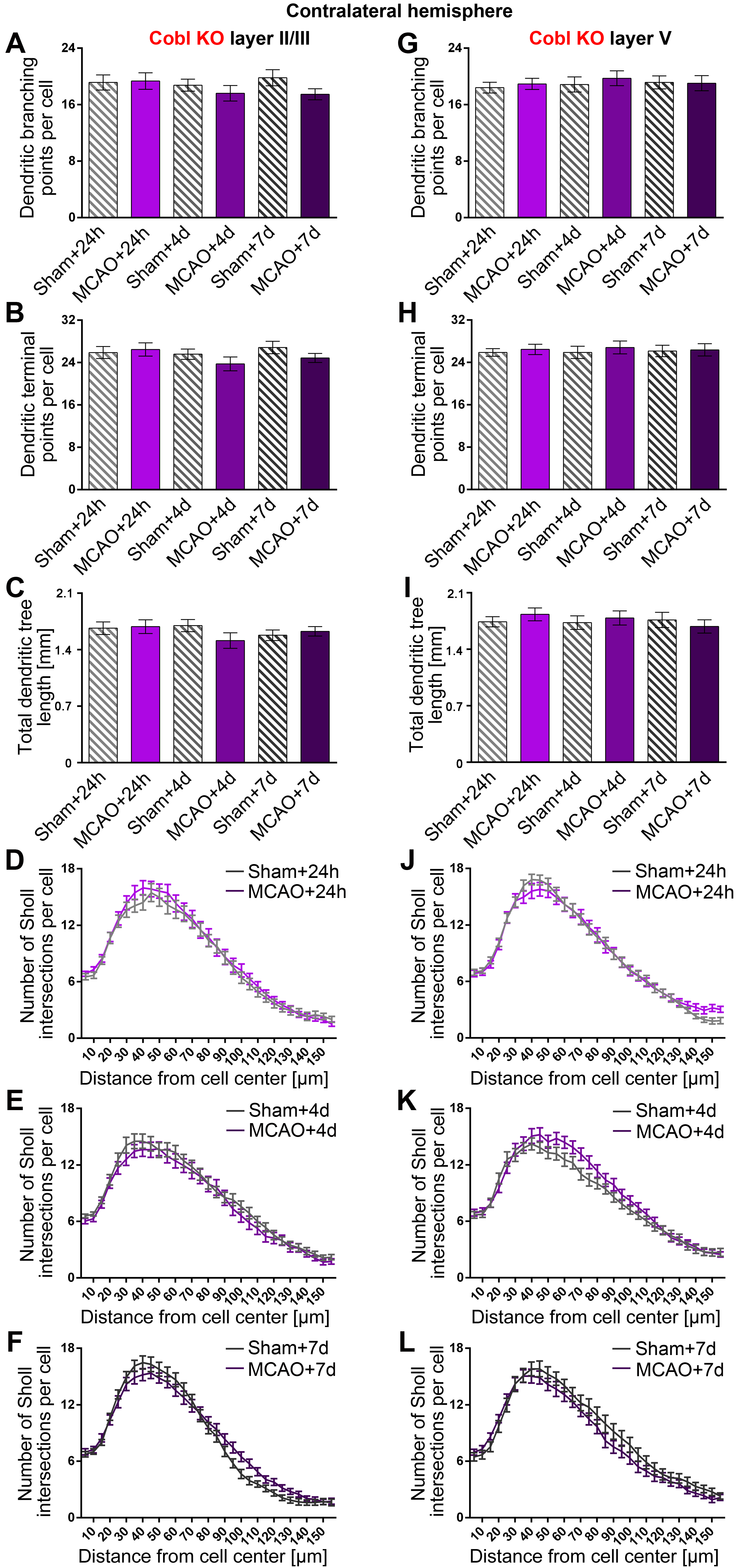

Supplement: S6 Fig — Cobl KO mice show no MCAO-induced dendritic alterations of the dendritic arbor at the contralateral side. A–L, Quantitative determinations of dendritic arborization parameters of layer II/III neurons (A–F) and layer V neurons (G–L) at the contralateral side in M1 of Cobl KO mice. Note that dendritic branching points (A, G), dendritic terminal points (B, H), total dendritic tree length (C, I), and Sholl intersections (D–F, J–L) at 24 hours, 4 days, and 7 days reperfusion times after 30-minute MCAO at the juxtaposed side all remained unchanged and similar to their corresponding sham controls. Layer II/III: nSham+24h = 28; nMCAO+24h = 32; nSham+4d = 33; nMCAO+4d = 28; nSham+7d = 31; nMCAO+7d = 36 neurons from 3 mice of each group. Layer V: nSham+24h = 34; nMCAO+24h = 33; nSham+4d = 28; nMCAO+4d = 34; nSham+7d = 26; nMCAO+7d = 27 neurons from 3 mice of each group. For corresponding ipsilateral data, see Fig 7. Data represent mean ± SEM. Statistical significances were calculated using 2-way ANOVA with Sidak posttest (all n.s.). The numerical data underlying this figure can be found in S13 Data. KO, knockout; MCAO, middle cerebral artery occlusion. (TIF) [file pbio.3001399.s006.tif]

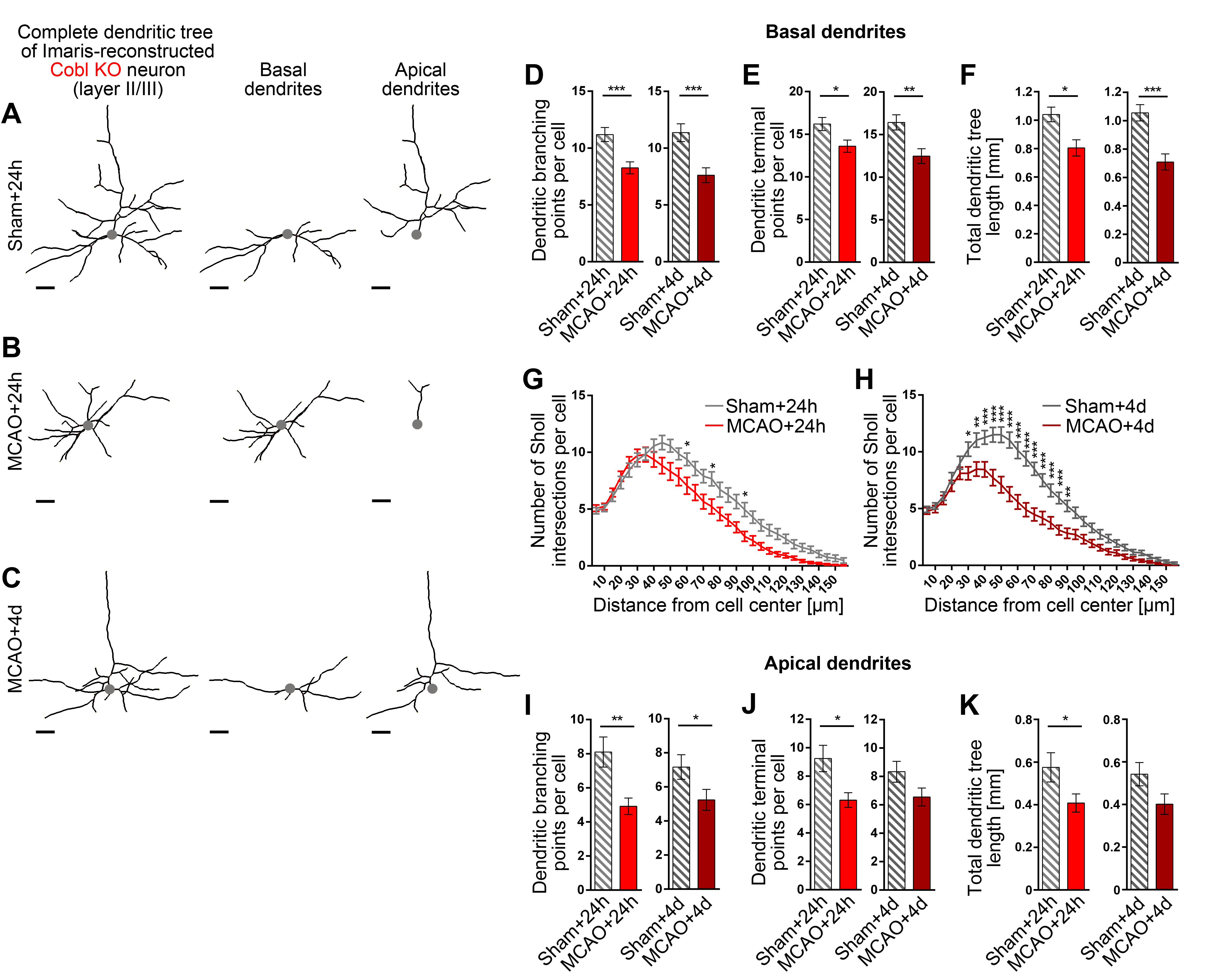

Supplement: S7 Fig — The defects in ipsilateral dendritic arbor repair observed in Cobl KO mice occur in both apical and basal dendrites of layer II/III pyramidal neurons of M1. A–C, Individual Imaris-reconstructed cells from Fig 7A–7C (right panels) showing ipsilateral layer II/III neurons from M1 of Cobl KO mice subjected to sham treatment (A) and 30-minute MCAO with 24 hours and 4 days reperfusion time (B, C), respectively, for differential analyses of basal (D–H) and apical dendritic parameters (I–K). The apical and basal dendritic parts are depicted in A–C. The somas are marked by a gray dot. Scale bars, 30 μm. D–K, Quantitative determinations of dendritic branching points, terminal points, total dendritic tree length, and Sholl intersections of basal dendrites (D–H) and related analyses for apical dendrites (I–K). Note that MCAO-induced defects occurring in both apical and basal dendrites 24 after MCAO, in contrast to WT (see Fig 5), do not show any recovery after 4 days reperfusion upon Cobl KO. nSham+24h = 21; nMCAO+24h = 35; nSham+4d = 30; nMCAO+4d = 29 neurons neurons from 3 mice for each MCAO and sham control group (ipsi). Quantitative data represent mean ± SEM. Statistical significance calculations, Mann–Whitney (D–F, I–K) and 2-way ANOVA with Sidak posttest for Sholl analysis (G, H), respectively. *P < 0.05; **P < 0.01; ***P < 0.001. The numerical data underlying this figure can be found in S14 Data. KO, knockout; MCAO, middle cerebral artery occlusion; WT, wild-type. (TIF) [file pbio.3001399.s007.tif]

Figure 1 - uncropped blots

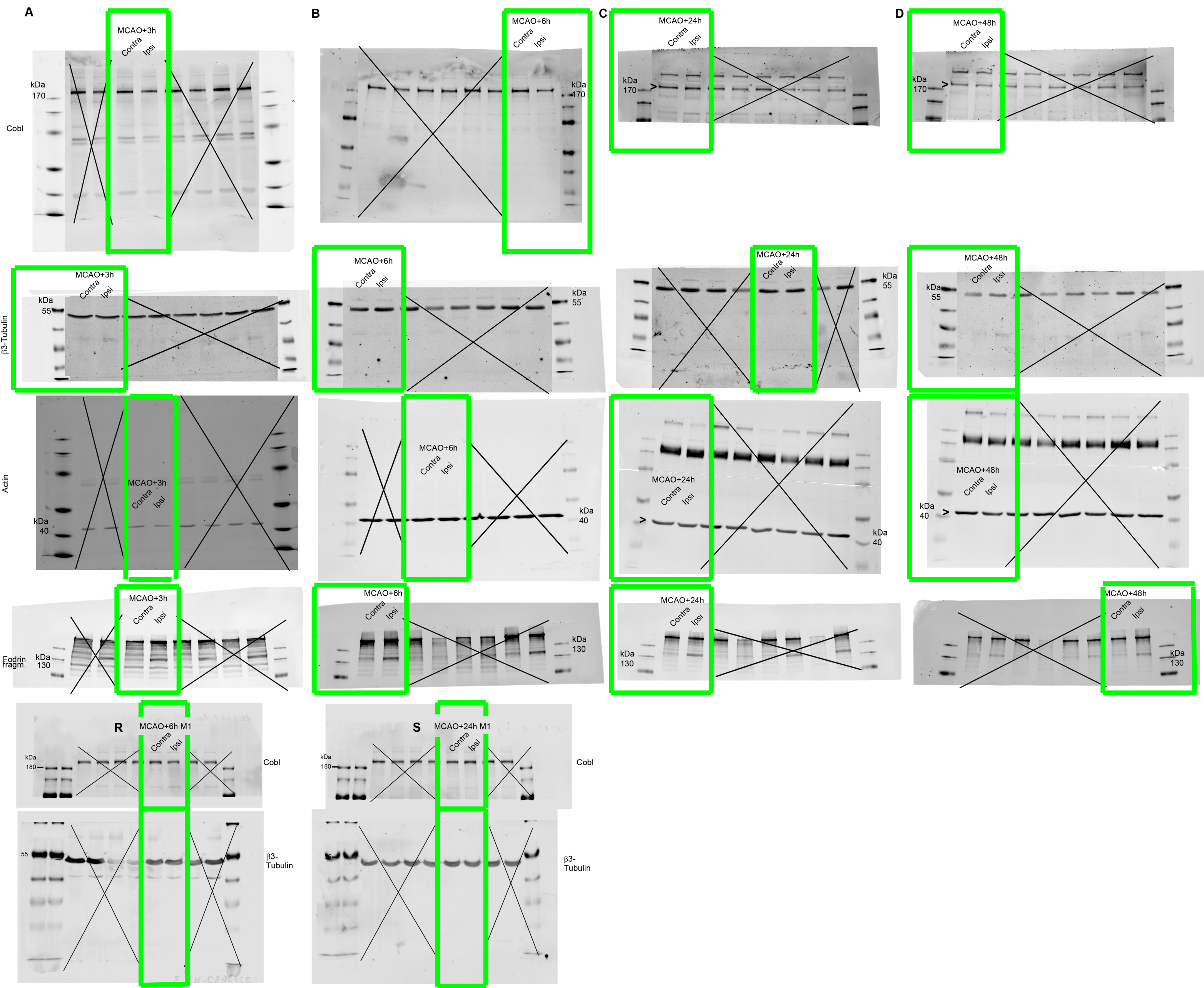

Figure 2 - uncropped blots

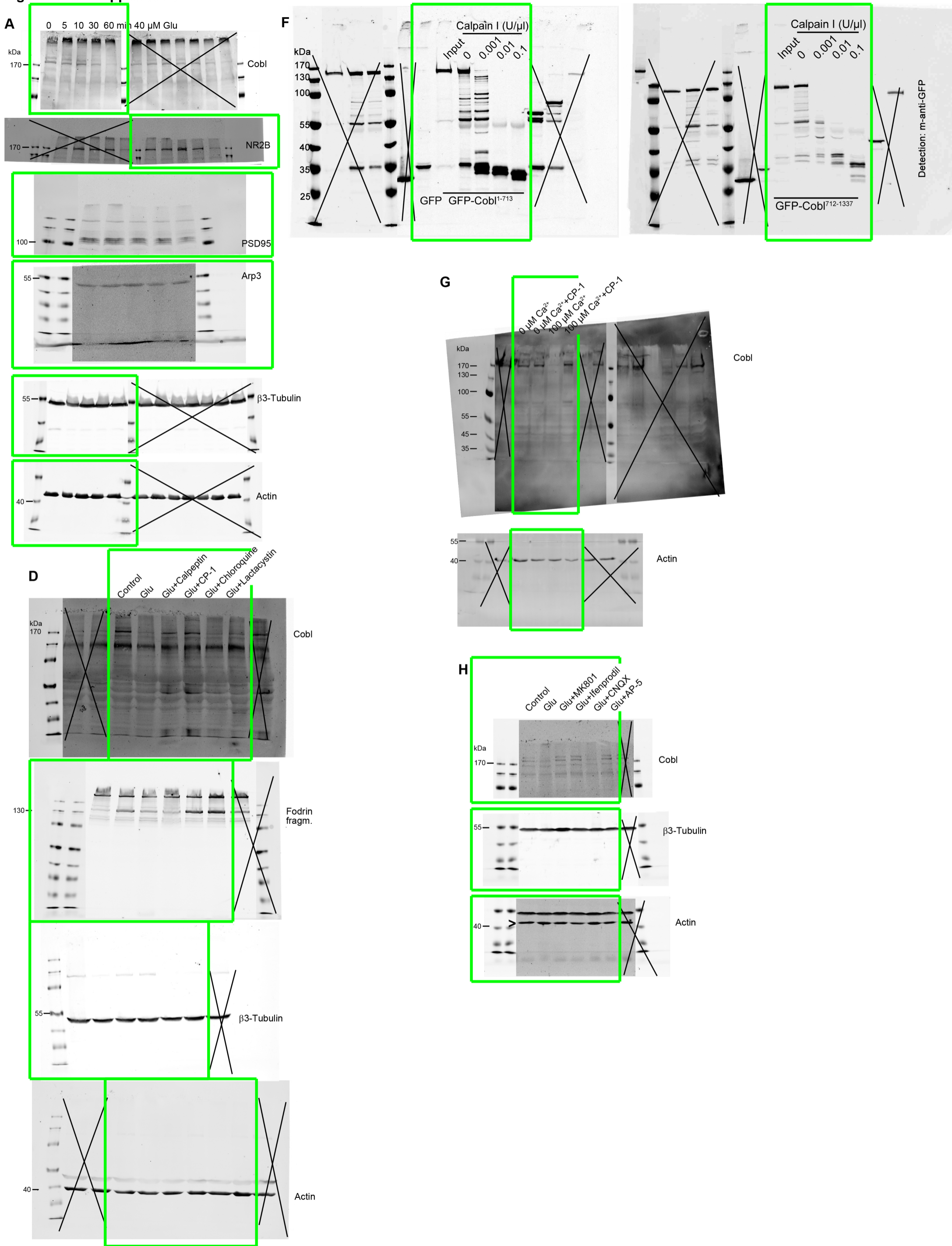

**Figure S1 - uncropped blots**

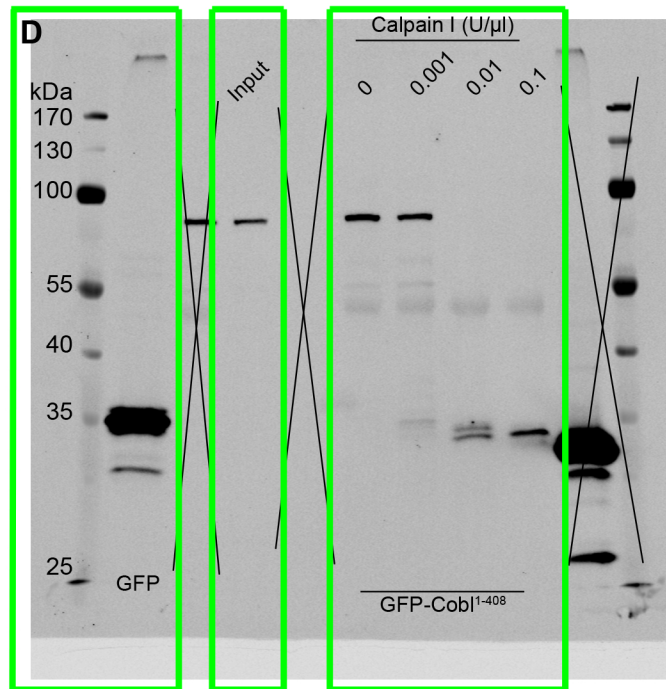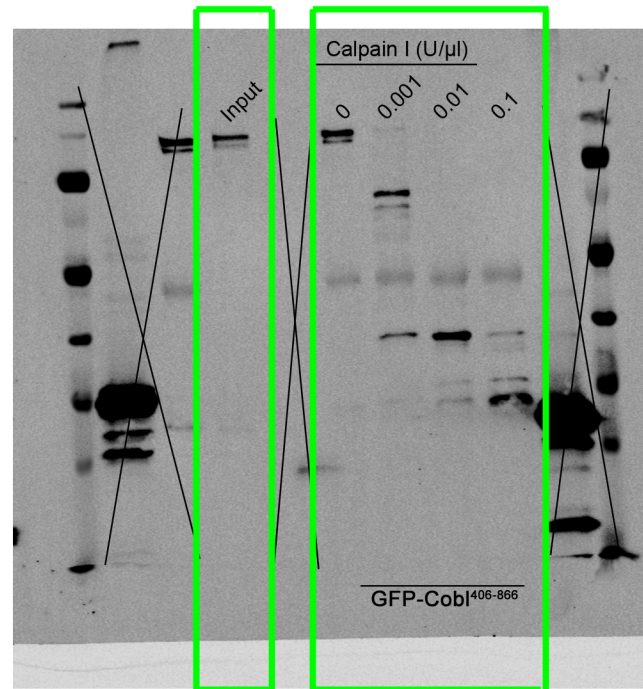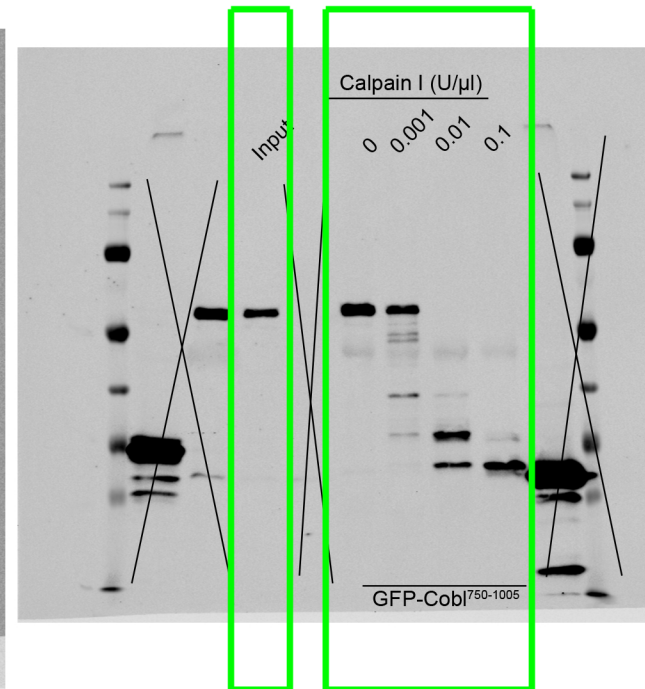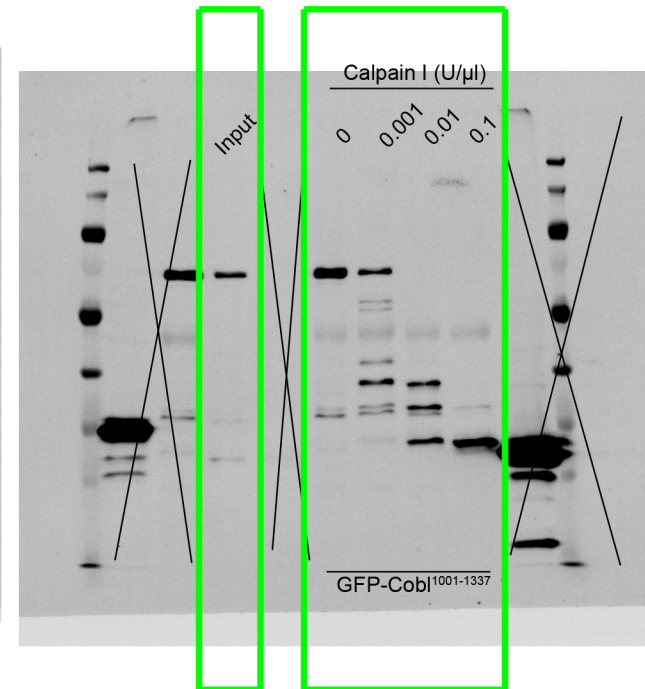

Supplement: S15 Data — This 3-page PDF contains all western blots shown in Figs 1 and 2 and S1 as an uncropped images. Lanes shown in the respective figure panel (see panel labeling) are framed in green, and lanes not shown are crossed out. Information on the samples (conditions) and on the immunodetections is labeled as in Figs 1 and 2 and S1. Note that some blots where physically cut for the multiple detects and thus do not include the full range of protein sizes (see standard lanes). (PDF) [file pbio.3001399.s022.pdf]
