# Supplementary material for: Poststroke dendritic arbor regrowth requires the actin nucleator Cobl
Source: PLoS Biol. 2021 Dec 13;19(12):e3001399. doi: 10.1371/journal.pbio.3001399 (PMC8699704; doi:10.1371/journal.pbio.3001399)
Supplement: Attachment — Submitted filename: Response_to_reviewers_11.doc [file pbio.3001399.s023.doc]

**Response to reviewers**

**Reviewer #1: Review of manuscript by Ji et al.**

In the present manuscript, the authors investigate molecular and anatomical phenomena taking place as a consequence of stroke. To do so, they employ the middle cerebral artery occlusion model in mice. They observe that in the penumbra region dendritic architecture of pyramidal neurons in layer II/III and V is transiently impaired. Moreover, they find that the protein levels of the actin nucleator Cobl are also transiently reduced after an excitotoxic/stroke event. Using a KO mouse for Cobl, they link the detected dendritic recovery to Cobl expression.

The work presented by the authors falls into a very interesting category of papers looking at more dynamics aspects of dendrites, which are not always brought into the spot light in favor of the more dynamic dendritic spines. However, some of the claims are not fully supported by their findings and should be better addressed before publication.

MAJOR

1- The in vitro experiments performed on cultured neurons were carried out using a relatively high concentration of glutamate (40µM). Is there a threshold level under which Cobl degradation does not happen and over which Cobl recovery does not happen?

It would be interesting, and easier to compare to the in vivo data and draw conclusions on the phenomenon, if some experiments involved the use of models better recapitulating the stroke conditions, such as glucose oxygen deprivation for instance.

As cited in the results part of the manuscript (Ankarcrona et al. 1995 *Neuron*), the excitotoxicity examinations were done according to well-established methods used in many high-ranking publications and were not particularly high for this purpose. Please see also compare e.g. the excitotoxicity studies of Xu et al., 2009 J. Neuroscience, in which even 100 µM glutamate was used, whereas we merely used 40 µM.

The exact threshold of glutamate leading to Cobl degradation is unknown. We know that one can obtain comparable data with 100 µM (our unpublished results). In general, key will certainly be a high enough resulting Ca^2+^ concentration for calpain activation, as we unveiled calpain to be the major player in Cobl degradation. Depending on isoform and calpain subunit, Ca^2+^ concentrations in the low-medium µM range would be sufficient. Under excitotoxicity conditions, these conditions will easily be reached.

For molecular mechanistic analyses, it is of course most powerful to address specifically whether a given newly identified phenomenon (here Cobl degradation) relates to what has already been understood as down-stream detrimental conditions caused by stroke. In ischemic stroke, the blood supply to a part of the brain is decreased, leading to dysfunction and disintegration of the brain tissue in that area. The established causal chain (ischemia => lack of energy => excitotoxicity due to glutamate release and Ca^2+^ influx) thus directly demands that for any molecular mechanistic understanding the focus must be on glutamate and Ca^2+^ as triggers for the degradation of Cobl. Thus, exactly these aspects were addressed in Fig. 2.

**As we have to acknowledge that also the introduction of the manuscript fell short of introducing these facts, this probably was hard to understand in the initially submitted manuscript. We hope that the reviewer will agree with us that the revised manuscript now makes it easier for non-expert readers to follow these lines of experimental design.**

2-as connections are considered to be vital for neuronal survival, how do the authors explain the no difference in stroke infarct shown in suppl fig 1? Considering the difference pointed out by the authors in dendritic remodeling of the two mouse lines, it would be expected to see an impact on neuronal survival derived from the differences in dendritic connections.

**We have tried to improve the descriptions and explanations in the revised manuscript:** First, as may be now more clearly described in the manuscript, the cell death that occurs in the striatum (infarct area) is obviously not related to the dendritic repair that happens in the penumbra, i.e. in a neighboring brain area that is at the forefront of repair/remapping once the excitotoxicity onslaught caused by 30 min MCAO is over. Second it is noteworthy that – as also described in the revised manuscript - that the initial ischemic stroke response in the penumbra (M1) is equal when WT mice are compared to *Cobl* KO mice (please compare Fig. 4 to revised Fig. 7 of the revised manuscript). In line, there also is no indication that neuronal survival is compromised over the entire life span of *Cobl* KO mice, as *Cobl* KO mice age quite normally and do not show any obvious degenerative phenotypes (Haag et al., 2018). Instead, it is exclusively the post-ischemic repair starting after day 1 that is different: full dendritic restoration upon day 4 in WT mice and complete lack of any dendritic repair in *Cobl* KO mice.

3-this aspect is particularly interesting as it is well known that mice are extremely resilient to stroke and after a few days their motor capacities can be very close to sham animals to the point of being indistinguishable. As such, are there any longer lasting impairments in the Cobl mice since they don't reestablish their connections as efficiently?

As described in the manuscript, dendritic arborization is still affected at day 4 and even at day 7 in *Cobl* KO mice, whereas WT mice already showed massive regrowth 4 d after MCAO.

However, if one evaluates the *Cobl* KO data carefully, it also becomes obvious that the difference of sham vs. MCAO animals becomes a bit smaller at day 7 in the layer II/III, which also repairs faster in WT (please see the p values of the MCAO data compared to the corresponding sham data declining from p<0.001 at 24 h and 4 d to p<0.01 and p<0.05 depending on parameter analyzed in *Cobl* KO mice; Fig. 7E-G). In the layer V, which is generally marked by a slower repair in WT mice (see e.g. Fig. 5K, day 4: dendritic branch points did not fully recover to sham levels but still showed some minor reduction (*)), this decrease in significant difference to sham at day 7 caused by a slight increase in arborization is less recognizable and only seen in the data of the total dendritic length and in the Sholl analyses in *Cobl* KO mice.

Thus, while MCAO defects in *Cobl* KO mice persist during all the time frame of repair studied, it may well be possible that, after some more weeks, also the dendritic arborization of cortical neurons in *Cobl* KO mice may eventually catch up with the WT repair.

**These aspects are now better described in the revised manuscript.**

4-What molecular pathways do the authors think are involved in the increased transcription of Cobl mRNA?

To our knowledge, nothing is known about the transcriptional control of Cobl. Thus, this question of the reviewer would certainly be an interesting subject of future studies.

Given the fact that we unveiled a glutamate and Ca^2+^-driven degradation of Cobl that would have to be sensed, it seems more than likely that these signals will also trigger the subsequent compensatory increase in Cobl gene expression. **This interesting point suggested by the reviewer is now covered in the discussion of the revised manuscript.**

5-Apical and basal dendrites of pyramidal neurons have been shown in several experimental settings to behave differently. Please show total dendritic length for the two compartments as distinct. In particular, as the Golgi technique very rarely allows reconstructions of the entire apical tree particularly when using relatively thin sections (like 100µm used in this study).

**As the reviewer requested, the revised manuscript now contains a dissected analysis of apical vs. basal dendrites.** This additional analysis was done for both the MCAO-mediated dendritic damage at 24 h as well as for the successful repair at 4 d in WT mice (**please see the** **newly added Fig. S4 in the revised manuscript**) and for the dendritic damage at 24 h and the lack of its repair after 4 days in *Cobl* KO animals (**please see the** **newly added Fig. S8 in the revised manuscript**).

Interestingly, the defects caused by 30 min MCAO in the M1 were equally strong at 24 h in apical as well as basal dendrites of both WT as well as *Cobl* KO mice. These findings are in line with a recent study of acute dendritic defects in rat, which also showed no general differences between apical and basal defects in rats (Hu et al., 2020). This also is in line with our findings that the repair process during the time between 24 h and 4 d after stroke induced by MCAO happened throughout the entire dendritic arbor in WT mice (**Fig. 4 and newly added Fig. S4**).

Thus, Cobl functions in dendritic repair seem not to be limited to some cellular subcompartments but are of importance throughout the entire dendritic arbor of cortical neurons.

MINOR

Relevant literature on restoring or dynamic sprouting of dendrites following a stroke should be better cited, a quick Pubmed search revealed, for instance, the recent Mauceri et al, PNAS 2020. But also several other papers based also on additional conditions such as seizures, strong learning paradigms etc showed plasticity of mature dendrites. Therefore, the authors should consider toning down the claims regarding novelty on plasticity of dendrites in mature neurons.

The revised manuscript now includes many more references, which describe the (mostly long-term) studies of dendritic arborization after ischemic stroke. **These also include the recent studies on acute defects of the dendritic arbor in the MCAO-induced penumbra in rats (Hu et al., 2020) and the study of Mauceri mentioned by the reviewer, which demonstrates that dendritic sprouting can be induced by VEGFD 24 h post-MCAO (Mauceri et al., 2020).**

**As the reviewer requested, the revised manuscript now also does not highlight anymore the contrast between the observed plasticity of dendrites and the textbook knowledge that dendrites are relatively static in adult brains.** The reviewer is of course right that there is some paradigms, that have been reported to result in changes of the dendritic arbor as such and not just in dendritic spine density or morphologies. Thus, it is of course clear that in principle also the dendritic arbor can change to some extent.

We have identified the exact time window of acute dendritic repair after ischemic stroke, identified the first critical molecular player for acute dendritic arbor repair by gene KO and studied its excitotoxicity-related aspects at the molecular and mechanistic level.

Why are the numbers of analyzed neurons occasionally very different? How was the sample size determined? (in example: nSham=60; nMCAO+6h=22; nMCAO+24h=11 neurons)

The reviewer is referring to Fig. 4. Sample sizes were not predetermined but taken as they were recordable from the approved animal n-number for the different conditions. Both groups (Sham and MCAO) contained 6 animals, the MCAO group was split to 3 animals MCAO+6h and 3 animals MCAO+24h in this rather exploratory study. As we conducted systematic sweeps across the brain samples in our blinded image recordings, the resulting n-numbers of neurons evaluated may differ.

The n-number for MCAO+24h, Layer II/III (n=11) is our lowest value for any neuromorphometric measurements reported (predefinition of data sets: they all definitively had to be larger than 10; ideally more than 20). Scientifically, the low n-number for this one data set was only accepted due to the following reasons:

i) the effects were clear and statistically significant (**Fig. 4G-J**),

ii) related data for MCAO+24h were obtained in layer V (here, with n=18) and were fully consistent (**Fig. 4K-N**),

and most importantly,

iii) the MCAO+24h data shown in Fig. 4 were later backed up by an independent animal cohort and a very consistent data set in the context of the recovery study addressing multiple time points (**Fig. 5**).

For these reasons, increasing the n-number for MCAO+24h/Layer II/III in Fig. 4G-J by subjecting further animals to MCAO to present more equal numbers was dismissed as unethical according to German animal welfare regulations.

The unusually high n number for sham controls in Fig. 4 finally is a result of pooling sham animals at different times post-Sham to reduce complexity - compare Fig. 5 and 6 (now Fig.7 in the revised manuscript) as well as Fig.S4 (now S5 in the revised manuscript) and S5 (now S7 in the revised manuscript) for separate data: sham 24h, 4d and 7d data are all similar, as there of course are no sham effects at any of the different time points.

Why not show the single points for the morphometric analyses as for the other experiment?

We show all individual data points in the multiple quantitative examinations of Fig. 1, Fig. 2 and FigS1 where n numbers are relatively low. The quantitative cellular analyses have higher n numbers but the data distribution in such analyses usually show deviations in absolute values that require relatively large axes because in parallel to accommodating the individual data points, the differences between the analyzed conditions also need to be visualized well. Thus, it is not helpful to depict all the individual data points of all the parameters of all the cells evaluated. We would also like to refer the reviewer to the fact that in our raw data file for each of the many quantitative panels of our manuscript the set of individual raw data is available.

Furthermore, we would like to refer the reviewer to one of our recent publications (Wolf et al., 2019 *Nat. Cell Biol.*), where the same issue arose. As also box plots and medians have their disadvantages, often are harder to fully understand for readers and in their standard form highlight rather uninteresting statistical significance range of p<0.05 (*), while most of our important data sets have p values of 0.01 or 0.001 (** and ***, respectively), we in this case with the editors decided to stay with the bar plots and for e.g. biochemical data show bar/dot plot overlays, as we did it here (see Fig. 1, 2, S1). We hope that is acceptable. Alternatively, we shall be happy to expand our (already extensive) supplementary information by alternative graphs of our quantitative data, in case the editors decide that due to formal reasons this is necessary for manuscript acceptance.

**Reviewer #2:**

The manuscript examines the role of an actin nucleator called Cobl in dendritic arbor regrowth after stroke. They show that ischemia transiently reduces Cobl levels at 3 and 6 hours after MCAO, which quickly becomes restored by 24h and is correlated with increased cortical dendritic arbor growth from days 2-4 post-stroke. They then show Cob1 KO mice lack dendritic arbor regrowth during the 2-4 day post stroke. Overall, I think the authors did a nice job presenting the biochemical and anatomical data, and provide interesting correlations between Cobl protein expression and lack of dendritic regrowth after stroke in Cobl KO mice. However, a direct demonstration that Cobl regulates dendritic growth in cortical pyramidal neurons after stroke, which is the primary conclusion of the paper, was unfortunately missing. Below are comments/queries that I hope the authors find helpful for improving the manuscript.

**The revised manuscript now demonstrates that Cobl regulates dendritic growth in cortical neurons explicitly by gain-of-function as well as loss-of-function (and rescue) analyses directly in these cells. Please see newly added Fig. 6 and the newly added supplementary figure (Fig. S6).**

Main Comments:

1)	Although I think the data presented in this manuscript is of high quality, there are some important parts missing that are needed to connect the overarching story. For example, the authors show a brain hemisphere wide reduction in Cobl at 3 and 6 hours, but not 24 or 48h post stroke.

The authors then show interesting correlations in layer 2/3 and 5 dendritic arbors in control and Cobl KO mice but there was never a direct demonstration that Cobl levels were altered specifically within ischemia affected cortical pyramidal neurons, nor was there a direct demonstration of Cobl induced growth in these cells. Consider for example that brain wide congenital knockout of Cobl could affect many other cells/factors that prevent dendritic growth or exacerbate degeneration in Cobl KO mice that are independent of neurons, such as the influence of vascular territory, blood flow, the blood brain barrier (edema) or neuro-glio-immune (oligodendrocyte, microglia interaction with neurons,

astrocyte swelling, monocyte invasion) interactions. Documenting blood flow changes in wild-type and Cobl KO mice during stroke induction is an excellent first step in understanding some of these other factors.

The reviewer is right, the fact that we found that Cobl clearly is a molecular target for excitotoxicity effects occurring upon stroke (reduced levels of Cobl 3 h and 6 h post-stroke), as determined by quantitative immunoblotting analyses of ipsilateral and contralateral brain parts, did not yet explicitly tell whether the reduction of Cobl really can also be detected in the penumbra, i.e. in the cortical neurons of the M1, after stroke. Although the reviewer will have to acknowledge that the fact that we were able to demonstrated that excitotoxicity processes triggered by Ca^2+^, calpain and the NMDA receptor in cortical neurons led to proteolytic Cobl degradation is a very strong argument for this. **Complying with the reviewers demands, the revised manuscript now contains quantitative immunoblotting data of specifically ipsilateral and contralateral M1 showing the significant decline of Cobl 6 h after MCAO and full recovery at 24 h (please see additional experimentation added to Fig. 1 (revised Fig. 1Q-U). The corroboration of our finding that Cobl shows an excitotoxicity-caused disintegration in M1 is therefore now linked directly to the post-stroke dendritic changes in M1 shown in Fig. 4,5 and 7 and the related supplementary figures, as the reviewer requested.**

**In fact, the revised manuscript now also explicitly demonstrates that also the second finding of Cobl expression dynamics, the transient upregulation of Cobl mRNA levels, correlated with the restoration of normal Cobl protein levels within 24 h, is observable in the M1 at both 6 h and 12 h after stroke (newly added experimentation to Fig. 3 and S2; please see Fig. 3E and Fig. S2E of the revised manuscript). Thus, also these observations at the molecular level are now more directly linked to the dendritic analyses in M1.**

The second general concern of the reviewer, the fact that our study lacks “*a direct demonstration of Cobl induced growth in these cells”* [i.e. in cortical neurons] is severe and well taken, too. Theoretically, it is of course possible that the lack of post-stroke dendritic repair we observed in *Cobl* KO mice is not caused by Cobl deficiency within these neurons but that instead blood supply differences, blood brain barrier effects, immunological signaling processes in the brain or body-wide and/or interactions with other cells may cause the observed defects in neuronal morphology by some unknown crosstalk with the nerve cells embedded into the tissue we analyzed. This theoretical risk is a very common caveat in gene KO studies at the whole animal level. Most KO studies fall short of addressing this, as often this is technically very challenging or close to impossible.

The reviewer suggested that “*Documenting blood flow changes in wild-type and Cobl KO mice during stroke induction is an excellent first step in understanding some of these other factors”.* Such measurements might indeed provide some insights into one of the many imaginable general processes by which neuronal morphology could theoretically be influenced indirectly instead of directly. **It would however be more elegant, if one could experimentally address the concern of the reviewer specifically and directly, i.e. address the acknowledged lack of a *direct demonstration of Cobl induced growth in these cells* instead of just excluding a single out of the many putative indirect explanations for the defects seen upon *Cobl* KO at the whole animal level. Obviously, such analyses have to erase biological complexity as much as possible and need to focus on cortical neurons explicitly.**

**The revised manuscript now includes not just one but even two of such experimental sets showing undoubtedly that Cobl “*induced growth in these cells [cortical neurons]*”, as the reviewer put it:**

**The newly added Fig. S6 shows a gain-of-function analysis in individual, dissociated cortical neurons and clearly demonstrates that Cobl promotes the dendritic growth in these cells (Fig. S6A-F).**

**Importantly, the newly added Fig. 6 furthermore reports a loss-of-function analysis in in individual, dissociated cortical neurons and clearly demonstrates that the actin nucleator Cobl is critical for exactly the dendritic parameter impaired in stroke repair in *Cobl* KO mice:** the numbers of dendritic branch points, the number of terminal points and the total dendritic length was observed to be reduced when Cobl was lacking in these cells. Sholl analyses similar to those conducted in the M1 tissue also showed the defects of Cobl deficiency throughout the dendritic arbor of these cells. Additional rescue experiments we included undoubtedly prove that the phenotypes observed are specifically due to Cobl deficiency in the cortical neurons (**please see the newly added Fig. 6 of the revised manuscript**).

2)	The present data implies, based on Golgi-Cox sampling at single timepoints, that dendritic arbors in wild-type mice can show massive regrowth from 2-4 days post-stroke. However, it is worth noting this data conflicts with previous literature although it seems that the authors are unaware of previous studies on this topic (see comment 3 below). For example, in vivo time lapse imaging of cortical dendritic arbors before and after stroke, found instances of surviving dendritic tips elongating or retracting (NOTE: tips skewed towards retraction) in the first week or two post-stroke, but neither showed de novo growth of branches.

The reviewer is probably referring to the beautiful work of Craig E. Brown et al. (2010 *J. Cereb. Blood Flow Metabol.*) showing a massive remodeling of dendrites in the somatosensory cortex at 2 weeks after photothrombic stroke but not at 4 and 6 weeks after stroke. Although not fully comparable with our study looking at much earlier time points (6 h and 24 h, damage; 4 d full net recovery in WT), these results are very interesting. **We thank the reviewer for making us revisit this paper again in detail. Brown et al. (2010) is now prominently discussed in the revised manuscript**.

In light of the Brown et al. data obtained by live imaging at 2 weeks after stroke, it seems possible that the massive induction of net dendritic growth and branch induction we observed at day 2 to day 4 may actually merely represent the tip of an iceberg in terms of the underlying cell biological process, as after reaching the net recovery at day 4, the dendritic growth and branching processes we described and identified to be Cobl-dependent may actually continue a while longer (at least until 2 weeks) because after day 4 it might be covered up by dendritic pruning processes operating in parallel. Therefore, Brown et al. (2020) may not have observed any net growth anymore two 2 weeks after stroke but they still detected high dendritic dynamics. The observed net growth into areas not so much affected by the infarct (distal) and a retraction of dendrites proximal to the infarct area (Brown et al., 2020) may then mostly be brought about by direction-dependent dendritic pruning. **As now discussed in the revised manuscript, together, these cellular response may be the basis for efficient remapping of neuronal circuits in the stroke-affected cortex.**

**In line with our data obtained by 30 min transient MCAO, also a recent study showed a massive dendritic impairment in the ipsilateral somatosensory cortex after stroke (at day 3 after strong but also transient MCAO (90 min)), which then had disappeared at the later time point analyzed in this study (21 days after ischemic stroke) (Hu et al., 2020).** Thus, in line with our observations and the critical time window (day 2-4) identified, also this recent study clearly showed a massive net growth and branching of the dendritic arbor sometime between day 3 and 21 after strong MCAO. In light of our more detailed data and in light of the data obtained by Brown et al. (2010) at 2 weeks after MCAO, it can be assumed that also the Hu et al. (2020) observations in fact represent a net repair during the early days after stroke. **This study is now also cited in the revised manuscript.**

Further, several previous Golgi-Cox studies have shown degeneration of cortical dendrities in the ipsilateral hemisphere after stroke. Therefore control data from past studies looks a lot like the Cobl KO mice, rather than the controls in the present study. Since it is established in the past literature and the present paper (see Contralateral data inSupp Fig 3) that distance from the infarct is a major factor in structural changes, I worry that the sampling in controls may be systematically different that the KO mice. Perhaps reporting structural changes as a scatterplot relative to distance would allay this concern.

We do not think that there is a gross conflict of our systematic data with the different and not always consistent and/or technically comparable reports in the literature. Instead, we would like to refer the reviewer to recently published data, which now is included in the revised manuscript (Hu et al., 2020).

1) Our results do not question reports of massive and maybe more permanent impairments very close or even inside of ischemic areas, as we did not study such damage zones and also did not use a paradigm of massive damage in the first place. Instead, we conducted a mild, transient MCAO corresponding to survivable human stroke not causing such strong damages (30 min MCAO) and we studied a part of the cortex that would have the chance to undergo remapping processes, i.e. is not marked by neurons barely surviving but is positioned far enough outside of the zone damaged by the infarct.

2) It may be noteworthy that also in our study the cortical layer V, i.e. the layer closer to the infarct area in the striatum, in fact was a little bit more weakly affected when compared to the more strongly affected layer II/III at MCAO+24h, which is further away from the infarct area in the striatum. Thus, dendritic damage of pyramidal cells of the M1 was not a simple function of distance to the infarct area but rather correlated with the neuronal circuits in the cortex.

3) We do not study any defects 2 or several weeks post-stroke but observed fast defects and also a relatively fast repair process that takes place in an important part of the penumbra, the M1.

4) The control data in WT mice at 24 h of course look like the *Cobl* KO data, as both genotypes show exactly the same defects. The differences arise at day 4 and 7 after MCAO, as which *Cobl* KO mice fail to show any repair of the 30-min-MCAO defects.

5) A systematic bias in either sampling and/or evaluation is very unlikely in our study, as – in part in contrast to literature studies - we have designed our study in form of blinded analyses and have conducted a systematic analysis yielding to sets of related and in part even independent animal cohorts and data sets, which importantly turned out to be very consistent and offer very detailed spatial but also temporal insights into dendritic post-stroke responses.

We show that even in ipsilateral cortical areas distal from the infarct in the striatum, the dendritic impairments cause by MCAO are massive, independent from the distance to the infarct and that Cobl was absolutely crucial for dendritic repair in both cortical layers, irrespective of their distance from the infarct areas and their speed of repair in WT.

3)	Some important statements about novelty and challenging dogmas are not well justified. For example in the abstract, the authors state that their findings "challenge the view that dendritic arbors are static". Similarly in the Introduction: "Surprisingly little is known about the fate of the normally very static dendritic arbor during stroke" and "These observations challenge the view that the dendritic arbor is static during adulthood." I'm confused by this, what dogma is being challenged here as I can think of at least 8 papers off the top of my head that describe dendritic arbor changes (Golgi-Cox, 2-photon imaging etc) after stroke, very similar to what is described here. A pubmed search easily reveals this literature. Instead the authors refer to a review from Koleske (2013) in multiple places (focused on molecular mechanisms of dendritic plasticity) that has just one brief paragraph (and just one citation) discussing dendiritc arbor plasticity after stroke. Bottom line is there is a bunch of literature out there on dendritic arbor remodeling after stroke that seems to have been overlooked by the authors.

**The revised manuscript now contains twelve literature references describing dendritic arbor examinations post stroke, which mostly were not included before.** The reviewer will, however, acknowledge that the majority of the literature is actually not directly comparable to our study on very early post-stroke dendritic arbor responses, as previous work (besides the work on spine dynamics) mostly focused on examining putative long-term changes weeks after stroke. In search for compensatory mechanisms, a significant portion of these studies even focused on putative dendritic changes at the contralateral side instead of focusing at the penumbra.

**In particular three papers are now more prominently discussed in the revised manuscript. These include the in vivo imaging study by Brown et al. (2010), the acute dendritic sprouting triggered by post-stroke VEGFD application (Mauceri et al., 2020) and the Golgi-Cox study of somatosensory neurons after strong MCAO examining day 3 and day 21 post stroke (Hu et al., 2020).**

**Also, we have erased references to the text book view that dendrites are more static from the revised manuscript,** because, as the reviewer correctly pointed out, there of course are reports that describe changes of the dendritic arbor during some, very diverse paradigms of physiological and pathophysiological changes in the brain. **As far as the explicitly criticized sentence of the introduction is concerned, the revised manuscript now reads:** “In contrast to dendritic spines and synapses, most dendrites and dendritic branches become stabilized at an age of about 20 days in mice (Koleske, 2013) and much less is known about the fate of the dendritic arbor during stroke when compared to dendritic spine dynamics.” Given the plethora of spine dynamic literature, this wording may be better than the previous text, which could have been misunderstood easily.

**We hope that the reviewer will be content with the changes made to the different parts of the revised manuscript.**

4)	Given the variance in data for 24 and 48h post-stroke, a sample size of 4 would seem insufficient to determine statistical significance, especially since earlier timepoints (3 and 6h) have double the sample (n=8 or 9).

As judged from the n-numbers and time points, the reviewer is referring to the quantitative biochemical data shown in Fig. 1. We agree with the reviewer that with an n number of 4 animals, the sample size would probably not be sufficient to pick up the deviation of Cobl expression levels we discovered (-25% at both 3h after stroke and 6 h after stroke (**Fig. 1E,F**)). Yet, the reviewer will acknowledge that the sample size should be high enough to serve as initial test for whether there could be any putative deviation (i.e. trend visible that may need to be explored further) or not at all. The results of these initial animal experiments were:

1. There clearly was no deviation of Cobl levels at ipsi when compared to contra at both 24 h or 48 h after stroke but the average Cobl levels were exactly the same at both 24 h and 48 h (**Fig. 1G,H**).

2. The data distribution is very regular around the mean in both cases and was not compromised by any single data points that could represent outliers (see individual data points shown in **Fig. 1G,H**).

3. Both independent data sets are fully consistent to each other (**Fig. 1G,H**).

4. At least for MCAO+48h the absolute scattering of the data points with about ±30% of the calculated mean was in exactly the same range as the distribution seen for MCAO+3h or MCAO+6h showing the statistically significantly reduced Cobl levels (both also around ±30% of the mean value) (**Fig. 1G,H**).

**Thus, considering the 3R rule (replace, reduce, refine) in animal experimentation, it was very hard to ethically justify why additional animals should be subjected to the suffering caused by MCAO** and then be killed late after MCAO to include even more data showing that – as expected and as already shown for 4 animals for two different time points that furthermore were fully consistent to each other - there are normal expression levels of Cobl levels in these cohorts of WT animals.

**To nevertheless try to address the concerns of the reviewer experimentally, we applied for an extended animal licence to be able to increase the n number of at least one time point during our resubmission work.** We were very happy to finally obtain the permission to conduct a few more MCAOs for this purpose from the responsible state office (*Thüringer Landesamt für Verbraucherschutz* animal licence UKJ-18-030a). We succeeded in inducing two strokes in these experiments and were thus able to increase the n number in the revised manuscript.

**The revised Fig. 1G (and Fig.1K and O) now shows the expanded data set with an n=6.** The result remained the same, absolutely now change of Cobl expression levels this late after stroke (**revised Fig. 1G**). The same applied to the β3-tubulin levels (**revised Fig. 1K**).

**However, the reviewer may be happy to see that now the (expected) elevation of the detection of the frodrin fragment at 24h post-stroke became statistically significant (revised Fig. 1O) and thereby highlights the difference to Cobl (see also Reviewer 4 point 4).**

5)	There is little information on the Cobl KO mouse. What cells in the brain normally express Cobl and is there are a complete or partial knockdown?

Cobl seems to be expressed in neurons in all areas of the brain (Ahuja et al., 2007; Haag et al., 2012). Functional data for neurons is thus far only available for early development of dissociated hippocampal neurons in culture (Ahuja et al., 2007 Cell) and in Purkinje cells of the cerebellum (Haag et al., 2012). The *Cobl* KO is a complete KO, as described (Haag et al., 2018).

**The revised manuscript describes these published data more extensively.** We hope that the reviewer agrees with us that the changes made improve the readability of the manuscript for readers that may not be so familiar with the actin nucleator Cobl.

6)	Figure 2H,I, the data appears to show that NMDA blockers not only prevent the degradation but lead to a significant increase in Cobl. I'm curious what the authors think is the explanation for this. Perhaps this warrants some comment in the discussion?

The observation of the reviewer is certainly interesting. It seems plausible that Cobl disintegration during the pathophysiological condition of stroke just mirrors in exaggerated form what under some physiological conditions (such as stronger synaptic transmission) may also occur. The apparent Cobl changes upon inhibition with MK801 or ifenprodil may mirror this.

However, the effects that the reviewer noticed are not statistically different from control. Thus, although maybe interesting for some readers interested in Ca^2+^-affected protein homeostasis, we had to abstain from describing this, as formally there is no such effect.

**Minor Comments:**

1)	The authors use the word "excitotoxicity" interchangeably for data from ischemic mice. I would recommend sticking with "ischemia-induced" and reserve "excitotoxicity-induced" for cell culture/slice experiments exposed to excess glutamate or NMDA.

**Changed according to the reviewer’s suggestion in the revised manuscript.**

2)	I think the manuscript could use some more editing as I had to read some sentences a couple of times to understand what was being said. One example is in the abstract: "In Cobl KO mice, the dendritic repair window determined to span day 2-4 post-stroke in WT strikingly passed without any dendritic regrowth but penumbral neurons remained at severe dendritic impairment states caused by stroke."

We thank the reviewer for his/her comment. **We broke the criticized sentence in the abstract into two separate and easy sentences and also made further changes to the revised manuscript.**

We hope that the reviewer will be content with the changes made and will agree with us that the revised manuscript now is easier to understand and read.

**Reviewer #3:**

The authors in the current study investigated the possible role of Cobl in dendritic arbor regrowth after ischemic stroke. They observed that the protein level of Cobl in the brain samples after MCAO was quickly reduced and then recovered likely due to its increased mRNA levels. In the cultured neurons they showed that Cobl reduction was caused the calpain-mediated proteolysis in a NMDAR-dependent manner. In the Cobl KO mice, the authors showed that in the repairing phase after ischemia, dendritic arbor regrowth was not found. The authors then concluded that Cobl is important for the dendritic arbor regrowth after ischemia.

Overall, this study unveils a novel function of Cobl in stroke. Several concerns need to be addressed before it could be published in this Journal.

1. It would be very helpful to map out the cleavage sites on Cobl by calpain. In their previous work (Hou W, Izadi M, Nemitz S, Haag N, Kessels MM, Qualmann B (2015) The Actin Nucleator Cobl Is Controlled by Calcium and Calmodulin. PLoS Biol 13 (9): e1002233. doi:10.1371/journal.pbio.1002233), they have constructed GFP-fused different fragments of Cobl.

**The revised manuscript now contains a more extensive analysis of the calpain cleavage sites using four additional fusion proteins spanning the entire Cobl protein** (Cobl^1-408^, Cobl^406-866^, Cobl^750-1005^ and Cobl^1001-1337^) **(please see the newly added Fig. S1D of the revised manuscript).**

In line with the results shown in the main figure (Fig. 2F), there obviously is a plethora of calpain cleavage sites within Cobl and these sites ensure that during excitotoxicity and ischemic conditions Cobl is effectively broken into smaller pieces and thereby cause a rapid and complete stop of Cobl functions, which involve both N and C terminal aspects.

2. Any reduction of Cobl in the layer II/III and V neurons after ischemia?

This apparently simple question is in fact is very difficult to clearly address experimentally, as polyclonal antibodies are of course unable to discriminate between peptides, protein fragments and intact proteins when used in IF studies.

**The revised manuscript now shows results obtained from new sets of animal experimentations with M1 preparations (new animal license UKJ-18-030a). Using dissected M1 (primary motor cortex) tissue pieces (see newly added Fig. 1Q) these experiments show that the Cobl degradation is not only seen in the full brain hemisphere including e.g. large parts of the MCAO-affected striatum and of the cortex in general (Fig. 1A-P) but that key findings can also directly be detected in M1 (newly added Fig. 1Q-U of revised manuscript).** Cobl degradation as a result of MCAO can be detected in the M1 6h post-stroke (**newly added Fig. 1R,T of revised manuscript**) and 24 h subsequent to stroke, Cobl levels were restored to normal levels in the M1 again (**newly added Fig. 1S,U of revised manuscript**).

It may in addition also be noteworthy that the experiments shown in Fig. 2 were done with preparations of cortical neurons. Such cultures of course mostly consist of pyramidal neurons building layer II/III and layer V, as putative layer I, IV or VI contributions in M1 are minor and layer IV is almost invisible even in microscopical examinations of M1. Using high-sensitivity fluorescence scanning by a Li-COR Odyssey, these Western blots of cortical cultures of neurons clearly and quantitatively demonstrated Cobl degradation effects similar to the cells in brain tissue (see the examinations shown in **Fig. 2**).

**In parallel, the revised manuscript addresses the second point of the observed dynamics of Cobl levels upon stroke, the compensatory upregulation of Cobl mRNA expression (6h and 12 h post-MCAO), which coincided with a full restoration of Cobl protein levels 24 h post-stroke.** **Also for this key finding of our study, we were able to demonstrate that an increase of Cobl mRNA also occurs in M1 at both 6 h and 12 h (newly added Fig. 3E and Fig. S2E in the revised manuscript).**

3. Experiments using the Cobl KO mice support a necessary role of Cobl in dendritic arbor regrowth after stroke. However, whether Cobl is sufficient for the dendritic arbor regrowth is not known.

The reviewer is right. Our work thus far was focused on the discovery that Cobl is critical for dendritic arborization in cortical neurons during repair processes after ischemia-induced defects in M1, i.e. on the loss-of-function phenotype of Cobl addressed by *Cobl* KO (and in the revised manuscript now also in individual cortical neurons with Cobl deficiency (newly added **Fig. 6**). The opposite, whether Cobl can power dendritic branching in gain-of-function experiments was not addressed.

**The revised manuscript now includes such Cobl gain-of-function experiments in cortical neurons. These experiments clearly demonstrated that Cobl promotes dendritic arborization (please see newly added Fig. S6).**

4. In the current stroke model, the M1 region was influenced. Whether Cobl KO mice showed more obvious neurological deficits associated with motor abilities after stroke?

Although it is not entirely clear from the literature which behavioral paradigm to study during the 4 days post-MCAO and how to exclude motorcoordinative functions of the cerebellum, it may indeed be interesting to correlate some putative motor deficits with the neuronal defects and the molecular changes involving Cobl seen in M1 as well as the Cobl-related mechanisms we identified. However, such an additional endeavor would require an application for yet another, in this case very much extended, animal license with a massive increase in animal numbers subjected to surgery and with a sharp rise of harm inflicted on the animals. We already had to apply for an increase in n numbers of mice to be subjected to MCAO-induced stroke due to the request of reviewer 2 to raise the n numbers in the immunoblotting analyses and to show Cobl decline and reexpression in specifically the tiny M1 tissue material. We were very happy when this new license was eventually granted and we could conduct our 2020/2021 resubmission work. In order to in addition even follow up some putative stroke-related motor deficits of both WT and *Cobl* KO mice at different times post-stroke, the animals in such cohorts would have to be encumbered even more than due to MCAO-induced stroke alone, as they would have to be forced through tasks addressing motor skills during the first days right after stroke.

Given the fact that it is obvious that recovery of disrupted cognitive abilities caused by ischemia-induced neuronal death of course requires that neighboring neurons (i.e. those in the penumbra) take over at least parts of the functions of the now lacking cells and that this requires a physical remapping, i.e. processes the cause dynamics in established circuits and build new connections, we currently are unsure whether such studies would be justifiable ethically and whether a license for such experiments would be granted by the responsible state office (*Thüringer Landesamt für Verbraucherschutz*).

**Reviewer #4:**

Dendritic spine plasticity/synaptic remodelling is known to occur in the penumbra region following stroke. This study by Ji et al. seeks to address remodelling of the dendritic arborisations themselves which, unlike spines structures, are thought to remain relatively static during adulthood. The authors previously discovered that cobl (Cordon-bleu) is an actin nucleation factor during developmental growth, specifying neuronal morphology and polarisation of some neuronal populations. The authors now reveal that cobl also regulates arbor-scale plasticity following stroke. Whilst the role of cobl as a regulator of dendritic growth was described some years ago, this represents the first description of a role in the adult, following a model of neuronal injury. The authors use a relatively mild, but clinically relevant, middle cerebral artery occlusion model of stroke to address this, which results in a striatal ischemic lesion. They describe that penumbral (motor cortex) dendritic arbour is rapidly, transiently degraded. This transience, or capacity for spontaneous arbor repair, is diminished in a cobl knockout mouse. Mechanistically they claim, via cell culture experiments with multiple pharmacological inhibitors, that loss of cobl following stroke, is well explained by glutamate, acting via NMDAR to increase calcium and calpain mediated proteolysis of cobl.

The labs of Qualmann and Kessels are well known for their beautiful work of characterizing cobl, and they collaborate here with an expert of cerebral ischemia, the Frahm lab. While the paper is overall well written and appears relatively well controlled, mechanistic insights are into how the dendrite remodelling occurs is not only lacking, but it not even clear whether some of the described stroke related molecular changes, occur in neurons. It appears that the paper is, unfortunately, not on the level of PLoS Biology. I have some specific comments below that may help the authors to prepare a submission to another journal.

**The revised manuscript now addresses the main concern voiced by the reviewer. It contains further experimentation besides the Cobl gene KO that show that dendritic remodeling by Cobl occurs in cortical neurons. Gain-of-function experiments show that the actin nucleator Cobl is able to bring about dendritic arborization. Loss-of-function and rescue experiments show that Cobl is a critical component in this process.**

**As both of these experimental sets were done in dissociated cultures and the manipulated cells are residing in a surrounding of WT cells, these experiments further more undoubtedly demonstrate that both findings represent Cobl functions directly inside of the individual cortical neurons manipulated.**

**Among other experimentation, the revised manuscript now also contains experimentation that shows the degradation of Cobl as well as the subsequent compensatory upregulation of mRNA levels restoring normal Cobl levels within the first 24 h post-stroke is occurring in the primary motor cortex, i.e. in the penumbra and brain region in which individual pyramidal neurons of both layer II/III and layer V have been analyzed in WT and *Cobl* KO mice and showed the impairment of restauration upon *Cobl* KO.**

Major points:

1) The brain lysate experiments would have greater significance if the penumbral region itself was probed for cobl levels, rather than the entire striatal/M1 hemispheric piece of tissue. The lack of change in beta-3-tubulin does not fit well with a change in M1 dendritic arbor, given that dendrites also contain beta-3-tubulin. This suggests that the lysates are too non-specific to evidence a) changes in penumbral cobl, and b) direct effect on neurons. Cobl effects could be non-neuronal. Clearly, microglia and astrocytes as well as oligodendrocytes could be affected. The latter is particularly important given that the cobl KO mouse is not a neuronal specific deletion. The same applies to where the cobl is later upregulated again.

The reviewer here raised two major and very fundamental concerns:

a) are the Cobl changes observable in the penumbra, such as in the motorcortex, in which the pyramidal neurons of both layer II/III and layer V were examined for their dendritic arborization over the time post-MCAO and

b) whether Cobl modulates the morphology of cortical neurons at all, as this has not been demonstrated, yet.

**We acknowledge that both concerns are valid and represent fundamental concerns. The revised manuscript now addresses both of these fundamental concerns by suitable experimentation.**

**a)** **That the identified changes of Cobl protein levels in the brains of MCAO animals (reduction 6 h post-stroke and the subsequent recovery to normal levels at 24 h post-stroke) occur in the primary motorcortex M1 is now shown by quantitative immunoblotting of specifically ipsilateral and contralateral M1 tissues (revised Fig. 1Q-U).**

**That also the identified upregulation of Cobl mRNA during the time leading to full restauration of Cobl protein levels occurs in M1 is now shown by qPCR analyses in the revised Fig. 3E and with an independent primer pair in the revised Fig. S2E (see also point number 2 of the reviewer and our answer below).**

The concern of the reviewer that a lack of change of beta-3-tubulin would not fit well with changes in dendritic arbor seems to be based on a misconception. Most proteins, including beta-3-tubulin are not degraded when dendrites are affected negatively, neither are they increased when dendrites grow or branch. Beta-tubulin just changes its polymerization state. The same is true for beta-actin, as we quantitatively show as well. Also in this case, the expression level is unchanged when compared to the intrinsic contralateral control.

**b)** **That Cobl indeed brings about dendritic arborization in cortical neurons and is critical for this process, is now shown by two additional data sets included in the revised manuscript:**

**i) Gain-of-function experiments show that the actin nucleator Cobl is able to bring about dendritic arborization (newly added Fig. S6).**

**ii) Loss-of-function and rescue experiments show that Cobl is a critical component in this process in cortical neurons (newly added Fig. 6).**

Both of these experimental sets were done in dissociated cortical cultures from WT animals. In these primary cultures, the manipulated neurons were thus residing in a surrounding of WT cells (containing all cells of the cortex, i.e. besides neurons also the microglia, astrocytes and oligodendrocytes of this brain parts the reviewer was concerned about).

**These newly added experiments therefore undoubtedly demonstrate that both the gain- and the loss-of-function phenotypes represent Cobl functions directly inside of the individual cortical neurons manipulated.**

2) Inclusion of immunostaining on brain tissue sections of cobl levels in neurons and non-neuronal cells is required to evidence that changes are occurring in penumbral neurons. Moreover, it would be important to understand where cobl is later upregulated again.

**For experimentation demonstrating that the observed chances indeed occur in the penumbra (M1) please see our response to point 1 above (newly added Fig. 1Q-U).**

**That also the identified upregulation of Cobl mRNA during the time leading to full restoration of Cobl protein levels occurs in M1 is now shown by qPCR analyses in Fig. 3 (newly added Fig. 3E) and in Fig. S2 (newly added Fig. S2E; corroboration with an independent primer pair) of the revised manuscript.**

Addressing such changes in the levels of intact Cobl by quantitative immunofluorescence analyses at different time points at the ipsi vs. contralateral sides of the motor cortex of WT animals subjected to MCAO vs. sham animals would not lead to reliable results, as polyclonal antibodies are unable to discriminate between binding of Cobl peptides or Cobl fragments and intact Cobl protein when used in IF studies.

3) Please include rescue of the cobl KO phenotype in cell culture to provide more mechanistic evidence.

We are not quite sure why the reviewer demands that the *Cobl* KO phenotypes we identified in dendritic branching in cortical neurons post-stroke need to be underscored by related rescue experiments. Other KO studies in highest-ranking journals describing some phenotypes in certain cells or tissues also do not do this. **Anyway, as already mentioned above, the revised manuscript now includes Cobl loss-of-function experiments in which the dendritic phenotypes caused by the deficiency of Cobl were all rescued to resupplying the neurons with Cobl (newly added Fig. 6).**

**As mentioned above, these results are furthermore supported by newly added gain-of-function data, which also show that Cobl plays an important role in dendritic arborization in cortical neurons (newly added Fig. S6).**

4) Figure 1. In 24h and 48hr timepoints there is only an n=4, and in the Fodrin fragment in particular, O and P appear higher in the ipsilateral side. Could the authors comment on choice of n-number for 24hr,48hr.

The reasons are the following: The eventual fading away of the fodrin fragment detection at some time point late after MCAO was not our focus in this study. An n number of 4 in quantitative immunoblotting analyses usually is high enough to show that there are no effects and the fodrin data 48 h after MCAO indeed showed just that. Thus, in consideration of the 3R principle, we stopped the subjection of more mice to MCAO for further 48h (and likewise also for further 24 h) data acquisition.

I appreciate that the point here is the early onset of fodrin-fragment, and lack of late cobl effect, but it would be good to include a statement on the maintenance of the fodrin fragment, for clarity when reading the graphs.

We thank the reviewer for his/her suggestion. The difference of the fodrin fragment and Cobl is indeed eye-catching. However, the elevated fodrin fragment at 24 h was not statistically significant in the previous manuscript and we therefore abstained from any comments about this.

**However, due to a demand to increase the number of independent animal experiments late after stroke from reviewer 2 (reviewer 2; point 4), we applied for an extended animal license to be able to increase the n number of at least one of the late time point during our resubmission work.** We were very happy to finally obtain the permission to conduct a few more MCAOs for this purpose from the responsible state office (*Thüringer Landesamt für Verbraucherschutz* animal license UKJ-18-030a). We succeeded in inducing two strokes in these additional experiments and were thus able to increase the n number for MCAO+24h in the revised manuscript to 6. As a consequence, while Cobl and ß3-tubulin, as expected, still did not show any changes, **the elevation of the fodrin fragment (despite high errors) now is not only more clearly visible but formally also statistically significant (p<0.05; *) (see revised Fig. 1O). We were therefore able to include a statement on the prolonged maintenance of the fodrin fragment in the revised manuscript, as the reviewer suggested**.

Figure legend: line 837 typo: "Representative".

We thank the reviewer for noticing this typo. **It was corrected in the revised manuscript.**

Minor Points:

Figure 2. It would be a positive addition to include some discussion on the lack of Arp3 change.

**The lack of changes of the Arp3 levels is now discussed along with the description of the results in the revised manuscript.** We hope that the reviewer is content with the addition made to the revised manuscript.

Supplementary 2B shows more variation within this Arp3 quantification, is there any correlation between particular samples and their NMDAR2B/PSD95 levels and Arp2/3 level?

The reviewer is probably referring to **Fig. S1B**. We are not quite sure what the background of the question of the reviewer is. The errors of the Arp3 quantification are in a quite normal range for biochemical examinations and for example roughly compare to those for PSD95 (**Fig. 2C**).

As the n-number was the same for the analyses of Cobl, PSD95, NR2B and Arp3, the slightly lower error ranges in the Cobl data in particular in the later stages (**Fig. 2B**; 30 min and 60 min) may relate to the fact that the Cobl decline was massive, i.e. the measured anti-Cobl intensities thus became very low. In contrast, Arp3 levels merely fluctuated around well-detectable control levels (**Fig. S1B**).

Correlations of Arp3 levels with either NMDAR2B or PSD95 levels were not recognizable.

Discussion

Line 359 typo- "restoration"

We thank the reviewer for finding this spelling error. **The typo has been chased by automatic searching and corrected 3times in the revised manuscript.**

Methods

Please include details on analgesics used for MCAO surgery.

**Done. Please see Materials and Methods of revised manuscript.**

Please include the concentration of antibodies used for immunoblotting.

**Done. Please see Materials and Methods of revised manuscript.**

Please include a statement as to rationale for concentrations of pharmacological inhibitors, antagonists and glutamate (were they described previously, was a dose-response experiment conducted?)

**The reagents were used according to established literature procedures cited in Materials and Methods and added to the reference list of the revised manuscript (Xu et al., 2009 *J. Neurosci.*).**

Line 405 - space missing before (

We thank the reviewer for finding this typo. **The typo has been corrected in the revised manuscript.**

Line 461 – typo

We thank the reviewer for finding this typo. **The typo has been corrected in the revised manuscript.**
