# Supplementary material for: Poststroke dendritic arbor regrowth requires the actin nucleator Cobl
Source: PLoS Biol. 2021 Dec 13;19(12):e3001399. doi: 10.1371/journal.pbio.3001399 (PMC8699704; doi:10.1371/journal.pbio.3001399)
Supplement: Attachment — Submitted filename: RE_to_Rev_ResubmREV1_5.doc [file pbio.3001399.s025.doc]

**Response to reviewers**

**Reviewer #1: -**

**We are happy to see that the reviewer 1 was content with the revised manuscript we submitted and thank the reviewer for his/her efforts in evaluating and improving our work.**

**Reviewer #2:** I appreciated the new data in Fig 1 showing changes in Cobl mRNA and protein expression specifically in the intact ipsilateral M1 (rather than whole hemisphere) which, is an important part of the story with respect to dendritic retraction and growth of pyramidal neurons. The authors have also done a much more thorough job placing the present findings in the context of past literature. In addition, the authors now provide more direct evidence between dendritic arbor remodelling and Cobl regulation with gain and loss of function experiments. Thus, the manuscript's primary conclusion has much stronger supporting data and is convincing. I fully support publishing this paper, although I have a few minor comments for the authors to consider.

**We thank the reviewer for his/her positive assessment of our revised manuscript and we shall be happy to address the minor points of the reviewer.**

-       The lack of observed changes in dendritic arbors in the contralateral hemisphere after MCAO is important (data in Supp Fig. 3 and 5) and is consistent with previous work from Johnston, Denizet et al., Cerebral Cortex, 2013.

**We thank the reviewer for this additional comment. The fact that the lack of any obvious contralateral remapping is consistent with previous work of Johnston et al. (2013) is now cited directly at the position where contralateral controls are described for the first time (Fig. 3; page 12).**

**Accordingly, the reference Johnston et al. (2013) has been added to the reference list of the revised manuscript.**

-       I was surprised the authors didn't combine Supp Fig 6 with main fig 6, since the data show growth promoting effect of Cobl overexpression (Supp Fig 6) while Cobl RNAi decreases dendritic branching. These 2 pieces of data when combined are quite strong, perhaps they could combine these into 1 main figure?

**We thank the reviewer for this suggestion. Fig. S6 has now been integrated into the revised main figure 6 (as Fig. 6A-F; former Fig. 6 panels now are G-M in the revised figures).**

-       Line 300-301: sentence is confusing "may not do so not only…"
 **Thank you for pointing out this confusing sentence. It has been improved in the revised manuscript.**

**Reviewer #3:** It is important to show the reduction of Cobl in the neurons of layers 11/111 of M1 where the dendritic arborization is reduced

This last remaining point of the reviewer in this review is a reiteration of the reviewer’s point #2 in the previous review: “2. Any reduction of Cobl in the layer II/III and V neurons after ischemia?”

Out of the suggestions by the editors how to approach this question, only immunostaining and Western blotting may be suitable to answer this scientific question because we demonstrated a calpain-dependent degradation of the Cobl protein downstream of NMDA and calcium signals caused by the excitotoxicity following ischemic stroke and not any decline of *Cobl* mRNA.

Western blot analyses of M1 we already added to the revised manuscript. Our quantitative evaluations of this tissue material from further animals we subjected to ischemic stroke clearly showed that (as already suggested from our analyses of whole central brain segments) also in the M1 one can observe an ipsilateral decline of Cobl at 6 h post-MCAO (**Fig. 1**). We furthermore showed that, also in the M1, this decline was compensated for in an effective manner 24 h after stroke (**Fig. 1**).

Technical trials showed that due to the already tiny piece of tissue representing the murine M1, it is futile to try to dissect this piece of tissue further into layer II/III and layer V on one side and layers I, IV and VI on the other side to address whether also in layer II/III and layer V, in which the *Cobl* KO phenotypes were shown, Cobl protein levels decline.

**Therefore, the newly revised manuscript now contains** **representative images and quantitative anti-Cobl immunofluorescence analyses of Cobl levels in both the layer II/III and layer V in the ipsilateral M1 in direct comparison to the contralateral M1 of animals subjected to ischemic stroke (Fig. 1V-X of the newly revised manuscript).**

In line with the massive proteolytic degradation of Cobl by a multitude of calpain sites (**Fig. 2** and additional data **Fig. S1D** in the revised manuscript), these studies clearly show that not only in the ipsilateral side of the full central brain segment (**Fig. 1A-H**), or in the ipsilateral M1 in total (**Fig. 1Q-U**) but **also in layers II/III and V of the ipsilateral M1 specifically, a clear decline of Cobl can be observed 6 h post-MCAO when compared to the contralateral M1 (Fig. 1V of the newly revised manuscript).**

**Quantitative assessments proved that this decline occurred in both layer II/III and in layer V. The decline** is in line with the massive proteolytic degradation of Cobl by a multitude of calpain sites (**Fig. 2** and additional data **Fig. S1D** in the revised manuscript) and **was in the same range as those observed for the ipsilateral, central block of the brain and the M1 in biochemical analyses (Fig. 1B,F,R,T). It was statistically significant for both layer II/III and layer V (Fig. 1W,X of the newly revised manuscript).**

We hope that the reviewer will acknowledge that the newly added data now clearly show that Cobl expression changes in response to ischemic stroke do not only occur widely in the ipsilateral hemisphere or in the M1 in total or in cortical neurons in culture but explicitly also in the pyramidal cell layers II/III and V, which we morphologically evaluated by Golgi staining in response to MCAO and/or *Cobl* KO, respectively.

**Reviewer #4:** The manuscript "Post-stroke dendritic arbor regrowth - a cortical repair process requiring the actin nucleator Cobl" improved in the revision process. I am sorry that I probably did not make it sufficiently clear when I wrote in my first comments "that it appears that the paper is, unfortunately, not on the level of PLoS Biology" and that "I have some specific comments below that may help the authors to prepare a submission to another journal".
In principle, I would expect for a PLoS Biology paper something on a different scale nowadays. For the claims the authors make they would need a KO model where Cobl is genetically ablated only in neurons and do the stroke work in such a model. This was clearly out of the scope of their work. And this is why I initially rejected the paper and gave some suggestions to improve the quality of the paper to submit it to another journal. Now the paper did improve. But it still is not on the level of a PLoS paper.  It just simply would in my opinion not be a PLoS Biology without such more rigorous analysis of Cobl function in vivo, as mentioned above.

We have shown the cortical defects in neuronal repair by *Cobl* gene KO. We have corroborated these findings by addressing both Cobl gain-of-function and Cobl loss-of-function effects in isolated cortical neurons in culture. It is known that Cobl is predominantly expressed in neurons and that the actin nucleator Cobl is important for morphological changes of hippocampal neurons and of cerebellar Purkinje cells during early development. Our finding that acute cortical dendritic arbor repair subsequent to ischemic stroke in adult mice requires the actin nucleator Cobl is in line with all of these findings.

Due to the above scientific arguments, a repetition of all our analyses with Cobl KO mice with mice merely lacking Cobl in neurons also would be hard to justify formally, as it would not be in line with the 3R principle in animal research, which at first and most prominently demands that the number of animals, which suffer in animal experiments (here the induction of ischemic stroke by MCAO), has to be strictly minimized.

**We are happy to see that the editors see this similarly and therefore also did not consider such analyses as required for publication in PLoS Biology.**

We would like to note that, in general, we do not think that complete gene KO is an invalid approach, which in all cases needs to be backed up by cell-specific KO, as the reviewer seems to imply.

Furthermore, it is worthwhile to consider that describing and studying gene KO or gene mutations at the whole animal level also may be closer to disease situations and the respective pathophysiologies than studying such defects in single cells or types of cells.
